# Eco-physiological adaptations, metabolomic profiles and genetic diversity across varied habitats in four medicinal plant species

**DOI:** 10.1186/s12870-025-07521-7

**Published:** 2025-11-14

**Authors:** Gamal E.A., Morsy A.A., Maher M. Shehata, Mohamed Ibrahim, Saleh H.A.

**Affiliations:** Department of Botany, Faculty of Science, Ain Shams University, Cairo, 11355 Egypt

**Keywords:** Habitat changes, Plant adaptation, Ecological drivers, Phytochemical constituents, Protein patterns

## Abstract

**Background:**

Plants are constantly in need of adapting to different environmental conditions and responses in many ways. The response of plants is different between different species to the same environmental factors. It is therefore important to fulfill more about how plants respond and adapt. This study aimed to analyze four different plant species collected from two different habitats. It focused on examining the responses of these species based on the composition of phytoconstituents, measurements of antioxidant compounds, and the expression level of cellular proteins.

**Results:**

Plant responses varied. Total phenolics varied in all plant species between different sites, as both *Tamarix aphylla* and *Erodium glaucophyllum* have high total phenolic in plant samples collected from Al Qalyubia and the revers was correct for *Zygophyllum coccineum* and *Haloxylon salicornicum*. The flavonoid is higher in samples collected from Al Qalyubia than from Al Suez in all plant species and the highest values recorded in samples of *E. glaucophyllum* (2.80 mg/g FW) and the lowest values recorded in *Z. coccineum* (0.19 and 0.173 mg/g FW). Correlation between plant Total phenols, Flavonoids, TAC, DPPH and soil analysis showed a significant negative correlation between DPPH and Total phenols. GC-MS analysis showed a remarkable variation in phytochemistry in plants from different location. The PCA analysis between soil and the GC-MS analysis and heatmap clustering correlation arranged plant in groups according to the similarity in phytoconstituents and soil, *Haloxylon salicornicum* showed the most distance between the samples from the same species and the short distance between samples of the same species was in *Zygophyllum coccineum* the data was matching with the TDS and EC analysis of soil samples. Genetic diversity was studied where total cellular proteins (TCPs) were extracted using 12% SDS-PAGE. The SDS-PAGE technique resolved clear and distinct protein bands ranging between 15 and 130 kDa. The results showed differential expression of multiple protein bands running at approximately 130, 70, 55, 50, 45, and 30 kDa in all studied samples, detected with various intensities. At the species-specific level, several unique protein bands were detected in some taxa but were barely detected or absent in others. Band scoring revealed a total of 38 protein bands with polymorphism percentage (P % = 73.68%) of 10 monomorphic and 28 polymorphic bands. The Euclidean distance tree revealed the differentiation of the eight samples into two main groups. Moreover, multivariate heatmap analysis was conducted and the results agreed with and affirmed the results of the cluster analysis.

**Conclusions:**

It could be suggested that the effect of the ecological drivers (viz. EC, TDS, Ca, Mg, Na, and SO_4_) on the metabolic activities, metabolomics of phytoconstituents, and the expression levels of cellular proteins influenced a differential behavior indicated through the results shown in the present study. This behavior could be linked and engaged with the protection of cellular metabolic activities and consistent protein expression against adverse climatic environmental conditions. This manuscript demonstrated the potential integration of ecological, physiological, and molecular analyses as a powerful strategy that can benefit different sectors of stockholders, including both academic and non-academic researchers, in sustaining the medicinal and economic significance of plant productivity.

**Supplementary Information:**

The online version contains supplementary material available at 10.1186/s12870-025-07521-7.

## Background

Abiotic stress on plants causes a variety of alterations to their physiology, molecular activity, and developmental processes. These changes may increase stress sensitivity, resistance, or tolerance [1]. There is a vast array of bioactive compounds found in plants. Most of these compounds are produced as secondary metabolites, which are compounds other than primary metabolites that are thought to help plants become more resilient to environmental stresses by interacting with their surroundings [2]. Plants’ ability to biosynthesize secondary metabolites is influenced by a variety of biotic and abiotic stressors in addition to genetic regulation [3]. By modifying the synthesis and upkeep of various collections of bioactive secondary metabolites, plants can adapt to ever-changing environmental conditions [4] such as water, light, temperature, soil, and chemicals. Secondary stresses are caused by negative abiotic variables including flooding or drought, light and temperature fluctuations, poor soil, or hazardous substances. These circumstances cause variations in the production of Secondary metabolites [5].

Most of the plant’s antioxidant components are phenolic, flavonoids, and diterpenes [6]. All sections of the plant, including the leaves, fruits, seeds, roots, and skin, contain free radicals. Secondary plant-derived metabolites, such as phenolic compounds, have a strong ability to eliminate these radicals [7]. In general, attention has been drawn to the antioxidant qualities and the impact of habitat on the quantity of secondary metabolites [8].Total phenolic content (TPC) and antioxidant activity were found to be inversely correlated with ambient temperature [9].

Soil is the most significant environmental component that stimulates secondary metabolites because it regulates the flow and accessibility of water, nutrients, and air [10]. Variations in temperature can affect the formation of secondary metabolites, with some molecules being more prevalent in particular temperature ranges [11]. Another important factor is the availability of water; for instance, drought stress can trigger the production of secondary metabolites that are involved in antioxidant defense and osmotic control [12].

Approximately 95% of Egypt is made up of desert ecosystems. The distinctive and distinctive xerophytic vegetation in this habitat provides the human population with necessities. Notwithstanding these advantages, recent history has documented challenges to its species and habitats. In addition to the aridity of the climate, human mismanagement of biological resources is the root cause of all problems [13]. In this respect, the vegetation of Eastern Desert of Egypt is under a huge human activity (mining, housing and industry) reflected in plant status [13]. From here come the selection of Suez locations and Al-Qalyubia to spotlight on the difference and the expected medicinal values of plants from different habitats.

The selection of the four plant species was based on their pharmaceutical and medicinal importance. *Tamarix aphlla* demonstrated antibacterial and anti-inflammatory qualities that were in line with their total phenolic and flavonoid contents, according to Hamed et al. [14]. These species were shown to have antibacterial action, antioxidant capacity, and a function in preventing cancer and other chronic illnesses [15]. Antihelmintic, diuretic, antioxidant, anti-hypertensive, antibacterial, and antifungal properties were reported for *Zygophyllum coccineum* L [16–18]. Known for its anti-inflammatory, antipyretic, analgesic, and antioxidant properties, *Haloxylon salicornicum* (Moq.) Bunge ex Boiss was introduced as a natural source of animal feed as a growth promoter and natural prophylactic antimicrobial, particularly in poultry and cattle farms, in place of chemical antimicrobials [17, 19]. The antibacterial, antioxidant, and anticancer properties of *Erodium glaucophyllum* (L.) have been established [20, 21].

However, so far, it is not clear how and whether the selected plant species in the presented study (based on their presence in two different locations) were significantly affected by recorded differences in climatic condition, Soil characters, and emerging circumstances. On bases of variations in the environmental conditions of various habitats will impact on plant metabolism, with notable consequences for the primary and secondary metabolites of the plant the aim of this study was to integrate the data outcomes of ecological, physiological, and molecular aspects in order to review and evaluate the impact(s) of recorded ecological drivers on the eight samples of *T. aphylla*,* Z. coccineum*,* E. glaucophyllum*, and *H. salicornicum* that were collected.

## Results

### Climatic conditions and soil analysis

**Fig. 1:**
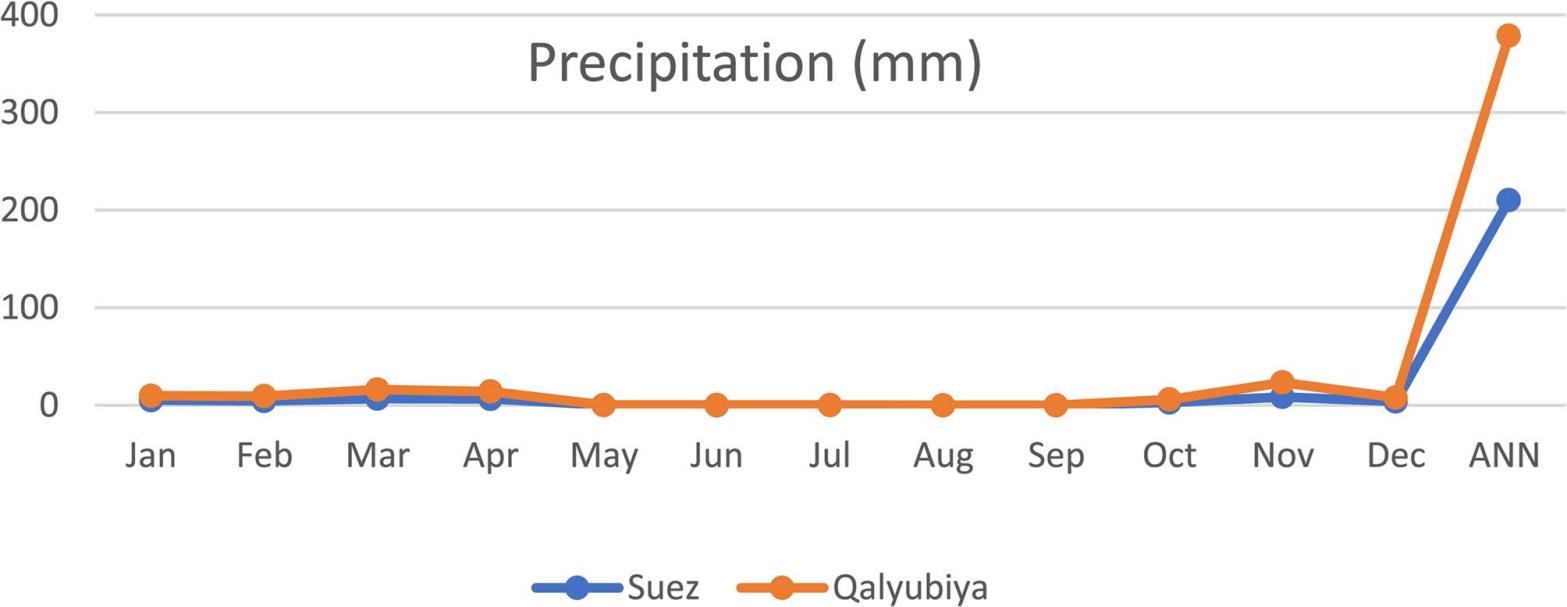
The mean precipitation (mm) of the study area averaged over the period of (2013–2023)

**Fig. 2:**
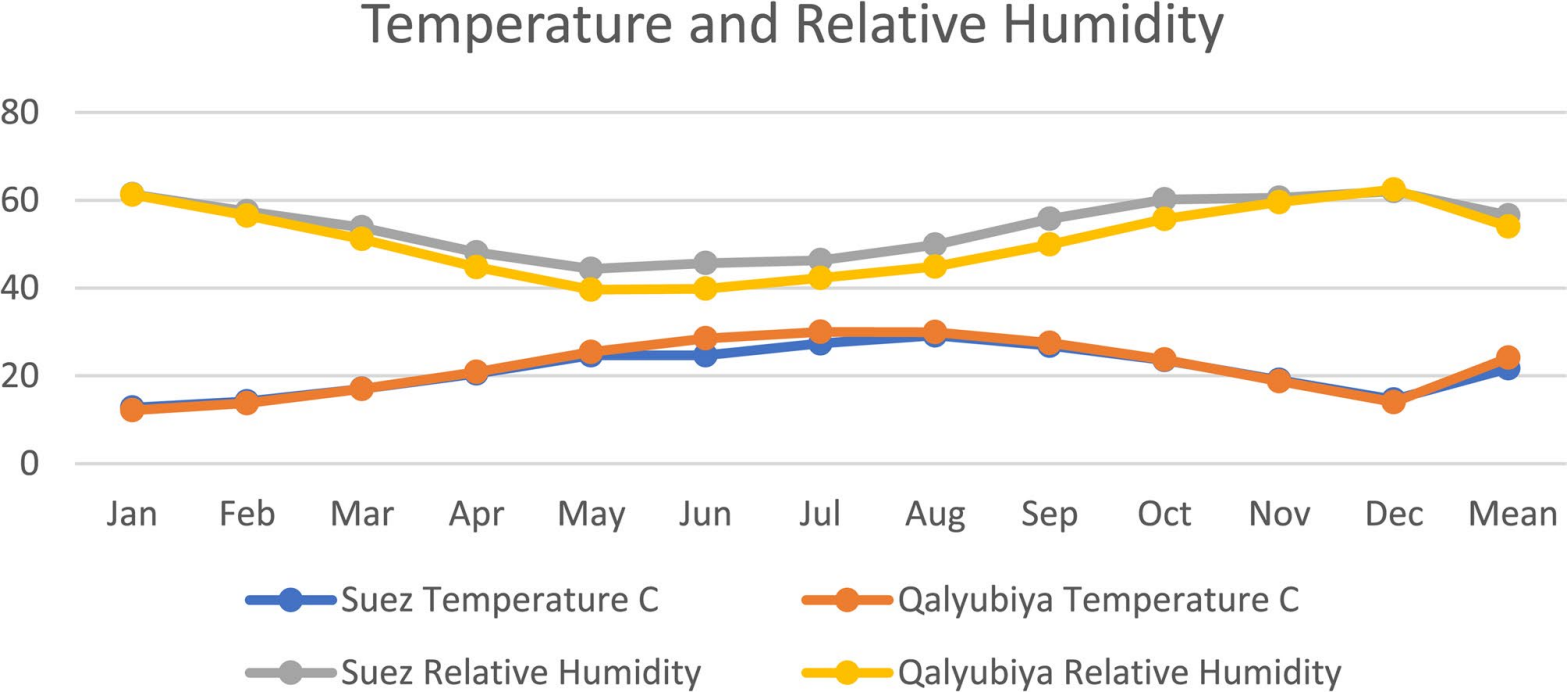
The mean temperature and relative humidity of the study area averaged over the period of (2013–2023)

**Fig. 3:**
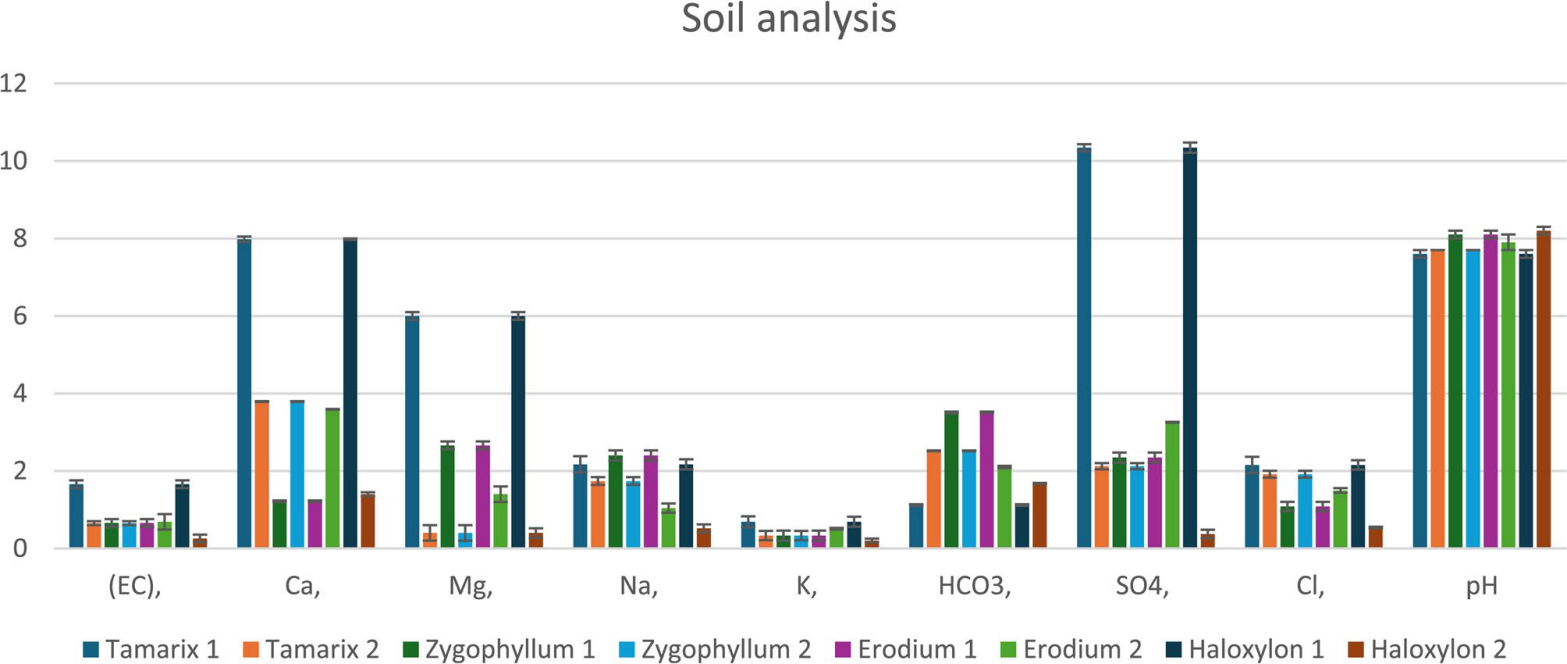
Measurements of the soil analyses of the two studied locations is presented in a bar chart showing the pH. EC, and minerals content (*meq/l*). Vertical bars represent ± SD

**Fig. 4:**
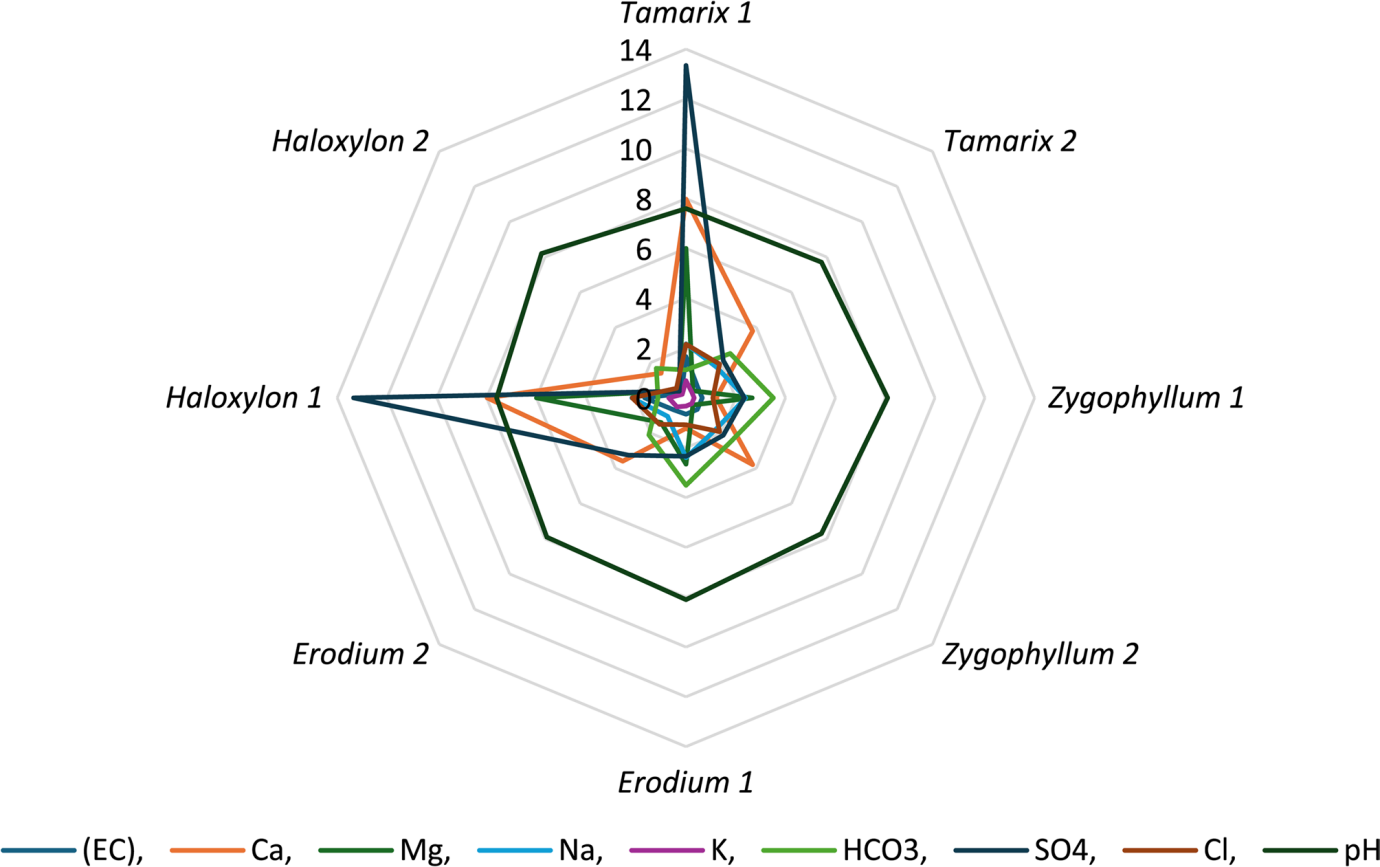
Radar graphs showing the variation in the soil analysis for different plants

Meteorological variables in terms of precipitation (Fig. 1), temperature and relative humidity (Fig. 2) were obtained from the NASA Prediction Of Worldwide Energy Resources (POWER) climate data portal, as shown downward in the materials and methods section. Each analysis was executed following all biostatistical needed requirements. The precipitation and temperature were higher in Al-Qalyubiya than Al-Suez but in reverse with humidity as it was higher in Suez than Al-Qalyubiya. All locations, where the soil samples were collected, were listed in details in Table 1. The texture of all soil samples (Table 2**)** showed the sandy texture of the soil collected from all location with variation in the ratio of size particles. Table 3; Fig. 3 showed the variation between different values of soil analysis. Q2 has the highest total dissolved salts (TDS) and electrical conductivity (EC) between the five locations (1061.9 mg/l and 1.656 ds/m respectively) and the lowest values were at S2 (141.1 mg/l and 0.256 ds/m respectively). Between the four plant species *H. salicornicum* has the highest difference in TDS and EC between the two location (Q2 and S2) and *Z. coccineum* has the lowest difference between the two locations in TDS and EC (Q1 and S1) (Figs. 3 and 4). 

**Table 1:** The locations and coordinates of the collected plant taxa supported with the associated plant species

Sites		Locations and coordinates	Collected plant species	Associated plant species
***1***	Q*1	**Al-Qalyubia Governorate** (Obour City) N 30 14 3 E 31 26 12	*Zygophyllum coccineum*,* Erodium glaucophyllum*	*Hyocyamus muticus*,* Aerva javanica*
	***Q2***	**Al-Qalyubia** Governorate (Obour City) N 30 14 5 E 31 26 10	*Haloxylon salicornicum*,* Tamarix aphylla*	*Hyocyamus muticus*
***2***	S*1	**Al-Suez Governorate** (Cairo-Suez desert road) N 30 06 31.9 E 31 40 43.9	*Erodium glaucophyllum*	*Anabasis setifera*,* Zilla spinosa*,* Zygophyllum coccineum*
	***S2***	**Al-Suez Governorate** (Cairo-Suez desert road) N 30 06 34.7 E 31 41 34.3	*Haloxylon salicornicum*	*Hyocyamus muticus*,* Zilla spinosa*,* Zygophyllum coccineum*
	***S3***	**Al-Suez Governorate** (Cairo-Suez desert road) N 30 13 07.6 E 31 21 47.1	*Tamarix aphylla*,* Zygophyllum coccineum*	*Zilla spinosa*,* Aerva javanica*

Q stands for Al-Qalyubia and S stands for Al-Suez Governorates, respectively

**Table 2:** Analysis of the texture of the soil(s) samples collected from indicated different locations

Sample	Particle size distribution (mm)					
	2 − 1	1-0.5.5.5.5	0.5 − 0.25	0.25 − 0.125	0.125 − 0.063	< 0.063
Q1	2.56	13. 81	**53.31**	23.12	5.47	1. 73
Q2	4.47	14.43	35.64	**34.24**	8.14	3.08
S1	12.47	**23.28**	32.64	19.76	8.80	3.05
S2	5.98	16.76	20.14	18.61	**29.67**	8.84
S3	**21.76**	**21.68**	19.87	16.40	9.79	**10.51**

**Table 3:** Soil analysis represented as means ± Sd (pH, EC, TDS, Ca^+2^, Mg^+^, Na^+^, K^+^, HCO_3_^−^, SO_4_^−−^, and Cl^−^)

Sample	pH	(EC), ds/m	(TDS), mg/l	Ca, meq/l	Mg, meq/l	Na, meq/l	K, meq/l	HCO_3_, meq/l	SO_4_, meq/l	Cl, meq/l
Q1	8.1 ± 0.1^b^	0.657 ± 0.02^b^	370.9 ± 3.5^b^	1.22 ± 0.1^a^	2.66 ± 0.13^c^	2.4 ± 0.13^d^	0.33 ± 0.02^b^	3.51 ± 0.13^e^	2.34 ± 0.12^b^	1.08 ± 0.1^b^
Q2	7.6 ± 0.1^a^	1.656 ± 0.07^c^	1061.9 ± 10.1^d^	7.98 ± 0.1^c^	6.00 ± 0.21^d^	2.17 ± 0.14^d^	0.69 ± 0.02^d^	1.12 ± 0.09^a^	13.34 ± 0.21^d^	2.16 ± 0.1
S1	7.9 ± 0.2^b^	0.686 ± 0.01^b^	406.6 ± 4.7^c^	3.59 ± 0.2^b^	1.40 ± 0.12^b^	1.04 ± 0.02^b^	0.51 ± 0.03^c^	2.10 ± 0.01^c^	3.26 ± 0.06^c^	1.49$\:\pm\:$0.09^c^
S2	8.2 ± 0.1^b^	0.256 ± 0.05^a^	141.1 ± 3.1^a^	1.40 ± 0.12^a^	0.40 ± 0.1^a^	0.52 ± 0.05^a^	0.21 ± 0.01^a^	1.68 ± 0.11^b^	0.37 ± 0.02^a^	0.54 ± 0.1^a^
S3	7.7 ± 0.05^a^	0.652 ± 0.01^b^	380.6 ± 5.8^b^	3.79 ± 0.2^b^	0.40 ± 0.1^a^	1.74 ± 0.12^c^	0.33 ± 0.01^b^	2.52 ± 0.08^d^	2.12 ± 0.09^b^	1.92 ± 0.2^d^

The values with same alphabetical letter in the same column are not significant

### Plant analysis

Total phenolics varied in all plant species between different sites (Table 4). Both *T. aphylla* and *E. glaucophyllum* have high total phenolic in plant samples collected from Al Qalyubia than Suez in reverse to *Z. coccineum* and *H. salicornicum* as the highest values were in plants collected from the Suez. The highest difference between samples from the two locations recorded in *T. aphylla* (5.59 and 1.04 mg/g FW) and the lowest recorded in *E. glaucophyllum* (3.07 and 2.94 mg/g FW). 

**Table 4:** Measurements of total phenols (mg/g FW), flavonoids (mg/g FW), TAC (µg/g FW), and DPPH (%) of different plant species from the studied two locations

Parameters Plant species	Total phenols (mg/g FW)	Flavonoids (mg/g FW)	TAC (µg/g FW)	DPPH (%)
*Tamarix 1*	4.59 ± 0.004	1.77 ± 0.39	617.6 ± 109.1	7.74 ± 1.36
*Tamarix 2*	1.04 ± 0.019*	0.47 ± 0.024*	333.07 ± 3.85*	40.9 ± 1.4*
*Zygophyllum 1*	0.73 ± 0.001	0.19 ± 0.006	85.63 ± 0.55	35.07 ± 0.65
*Zygophyllum 2*	2.05 ± 0.26*	0.173 ± 0.0015	59 ± 1.7*	41.63 ± 0.35*
*Erodium 1*	3.07 ± 0.007	2.80 ± 0.06	650.07 ± 70.75	12.1 ± 2.1
*Erodium 2*	2.94 ± 0.003	1.33 ± 0.016*	707.43 ± 19.25	30.7 ± 0.1*
*Haloxylon 1*	0.38 ± 0.009	0.27 ± 0.0015	201.63 ± 20.55	35.3 ± 0.9
*Haloxylon 2*	0.55 ± 0.012*	0.22 ± 0.007*	264.13 ± 11. 15*	35.9 ± 0.5
LSD	**0.074**	**0.023**	**38.65**	**0.9**

1; plant collected from Al-Qalyubia. 2; plants collected from Al-Suez LSD stands for least significant difference

*The asterisk denotes for the mean difference is significant at p-value 0.05 level (the comparison between the same species from different locations)

Flavonoids concentration in all plant species (Table 4) showed that flavonoids are higher in samples collected from Al-Qalyubia than from Al-Suez in all plant species and the highest values recorded in samples of *E. glaucophyllum* (2.80 mg/g FW) and the lowest values recorded in *Z. coccineum* (0.19 and 0.173 mg/g FW).

Total antioxidant capacity (Table 4) higher values recorded in *E. glaucophyllum* (650.07 and 707.43 µg/g FW) and lowest values were recorded at *Z. coccineum* (85.63 and 59 µg/g FW). Both *T. aphylla* and *Z. coccineum* have higher values of TAC in samples from Al-Qalyubia than Al-Suez and the revers was for *E. glaucophyllum* and *H. salicornicum*.

DPPH radical scavenging assay (Table 4) highest values were recorded in *T. aphylla* and *Z. coccineum* samples from Al-Suez (40.9 and 41.63**%** respectively), while the lowest value was recorded in *T. aphylla* from Al-Qalyubia (7.74%).

**Fig. 5:**
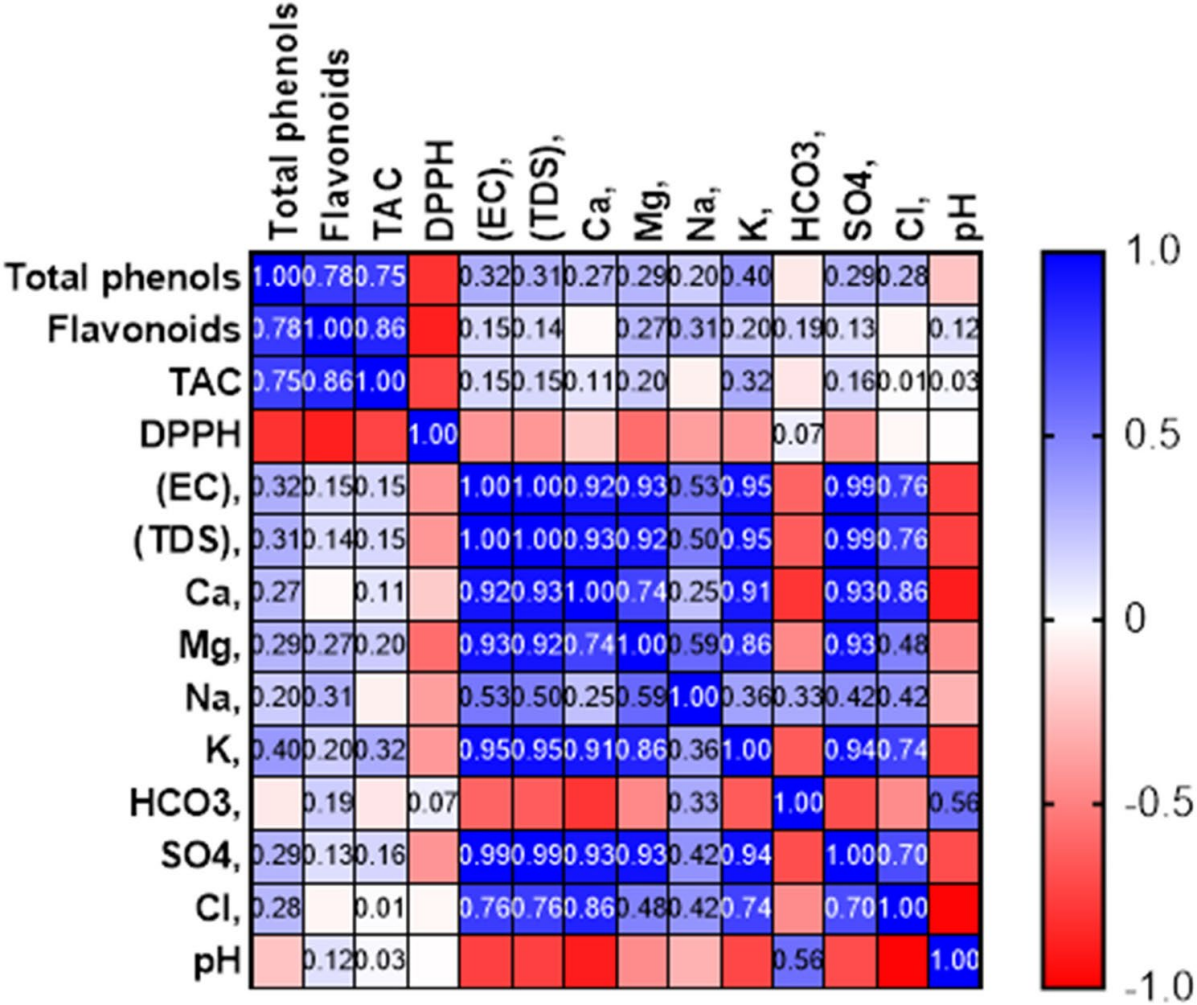
Pearson correlation between plant total phenols, Flavonoids, TAC, DPPH, and soil analysis

Pearson correlation between plant Total phenols, Flavonoids, TAC, DPPH, and soil analysis (Fig. 5; Table 4) showed a significant negative correlation between DPPH and soil EC. Significant positive correlation between TAC and Flavonoids was found. Both EC and TDS of soil showed many significant positive correlations with soil cations and anions.

Pearson correlation between Total phenols, Flavenoids, TAC, DPPH and soil factors was shown (Fig. 5). A positive correlation between total phenols, Flavonoids, and TAC but a negative correlation with DPPH was observed. Also, there is a strong positive correlation between EC, TDS, Ca, K, Cl, SO_4_, and Mg at the same time positive correlation between Total Phenols and different soil factors.

GCmass analysis of chloroform extracts from four plants showed varying results across species (Tables S1-S4). Among all the identified constituents, only 33 were found in more than one species (Fig. 5). Pentasiloxane, dodecamethyl was the only concerted compound in all four plants but not in all sites, followed by Glycerol, 3TMS derivative, Octacosane, 1-iodo- and Eicosane were found in three plant species and the other 29 constituent found in two species. GC-MS for *Tamarix aphylla* (Table S1) showed identification of 58 compounds, most of them were alkanes, with 15 common between the two sites; samples of Al-Qalyubia had 37 compounds showing an increase in phenolic compounds as Silanol, trimethyl, while Al-Suez had 36 compounds most of them identified as hydrocarbon molecules as Octadecane;3-ethyl-5-(2-ethylbutyl).

In *Zygophyllum coccineum*, 67 compounds had been revealed (Table S2), with commonly 12 compounds across the two sites. Alkanes was the main category followed by fatty acids and fatty alcohol in addition to phenolics. Al-Qalyubia showed identification of 42 compounds with a higher presence of fatty acids e.g. Palmitic Acid, TMS derivative, whereas 36 compounds identified in samples from Al-Suez. For *Erodium glaucophyllum* (Table S3), 45 compounds were identified, with majority of alkanes, and only six compounds were common in the samples from the two sites. In samples from Al-Qalyubia, 27 compounds were identified, whereas 24 compounds were identified in samples from Al-Suez with more phenolics in Al-Qalyubia. *Haloxylon salicornicum* (Table S4) exhibited 76 compounds, with only seven common compounds. Al-Qalyubia samples had been revealed 44 compounds among them sugar compounds such as D- (-)-Tagatofuranose, which were not detected in Al-Suez.

**Fig. 6:**
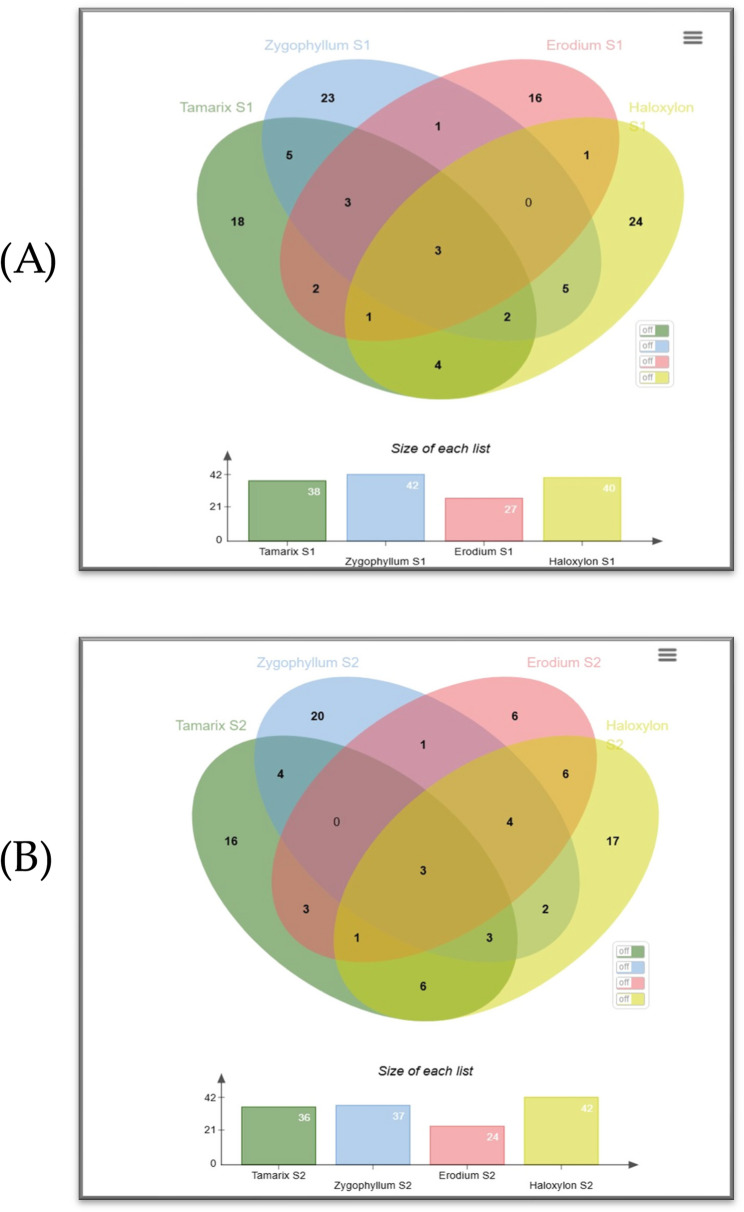
Venn diagram for the Gc-mass analysis of studied plant taxa. **A** for plants collected from Al-Qalyubia Governorate and (**B**) for plants collected from Al-Suez Governorate

Venn diagram (Fig. 6) shows the resemblance between plants from the same locations, the number of common identified phytochemicals in common between plants is higher in plants collected from Al-Suez government than plants collected from Al-Qalyubia government.

**Fig. 7:**
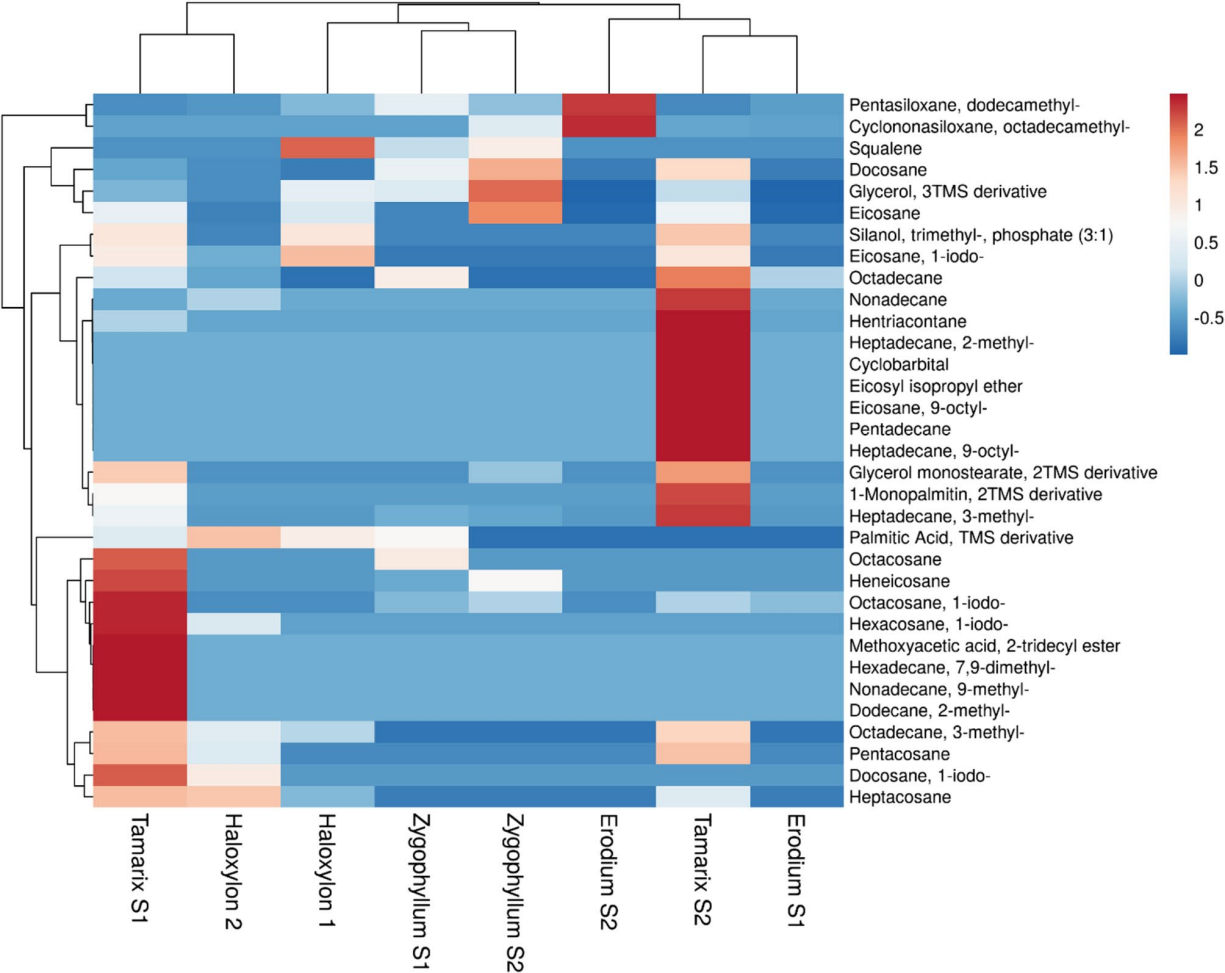
Multivariate heatmap clustering was constructed using correlation distance and average linkage based on the common 33 phytoconstituents detected in the four studied plant species

Cluster analysis was conducted using common 33 phytochemistry in the selected species (Fig. 7) distinguishing the 8 plants into 3 main groups. Group one compresses *Erodium*1, *Erodium* 2 and *Tamarix* 2, group two include the two *Zygophyllum* and *Haloxylon*1 and group three included *Haloxylon* 1 and *Tamarix* 1. Also, samples of *Tamarix* showed the most distance between the samples from the same species and the short distance between samples of the same species was in *Z. coccineum*; the data was matching with the TDS and EC analysis of soil samples.

**Fig. 8:**
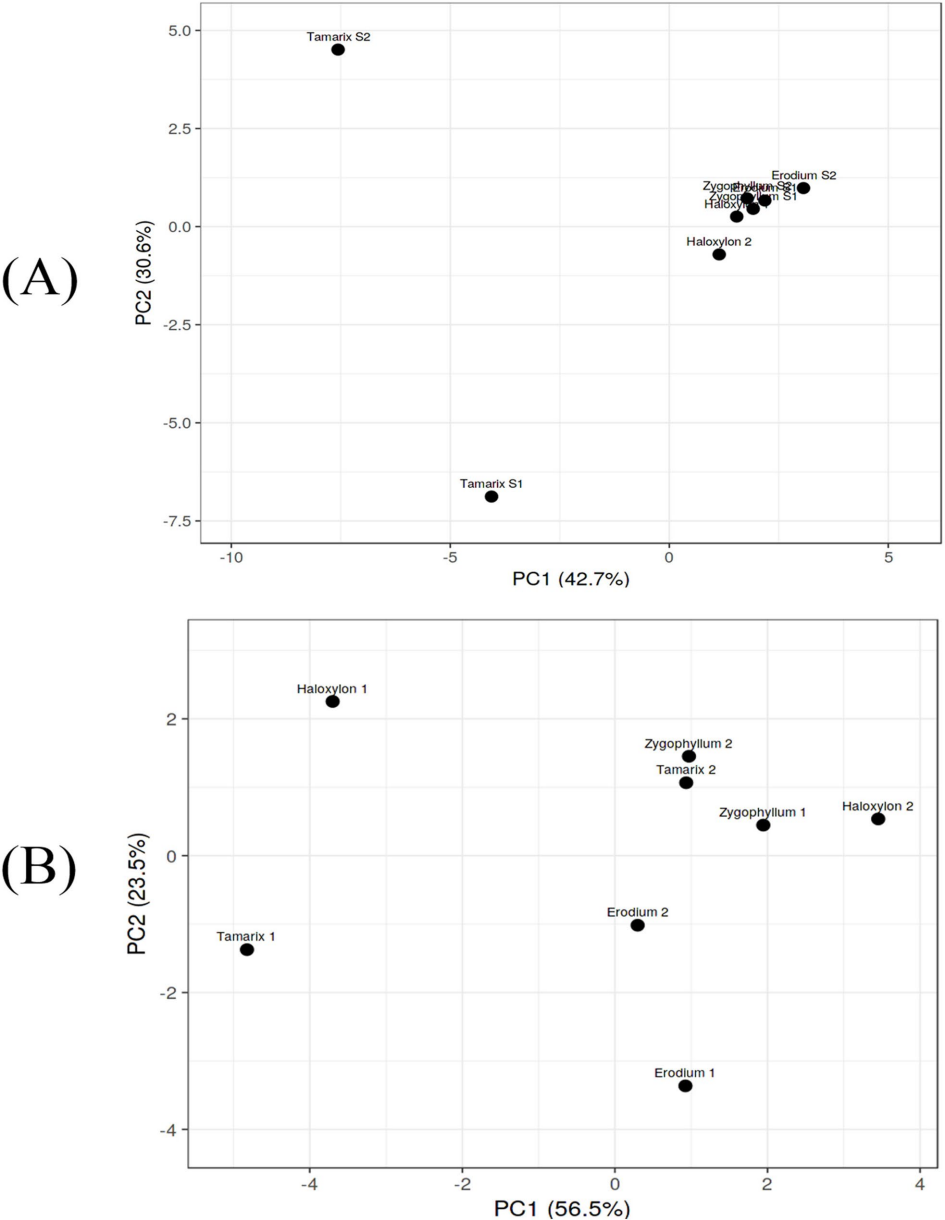
**A** PCA analysis for the common 33 phytoconstituent detected from GC-MS analysis of the plant samples. **B** PCA analysis of the combined measurements of the total phenolics, flavonoids, DPPH, TAC, and soil analysis

PCA test was applied two times; one was for the common 33 phytoconstituents detected in the selected species (Fig. 8a; Table 5) and the second was for the physiological parameters with soil analysis (Fig. 8b; Table 6). The PCA for the common phytochemistry revealed that the first two PCA are counted for 73% of the total variance (Table 5). From the selected 33 compound only five compounds with positive correlation in PC1. The result of the PCA analysis indicate the cluster result as the higher distance between samples from the same species was for *Tamarix*. The results of principal component analysis (PCA) for physiological parameters and soil revealed that the PC1 and PC2 are accounted for 80% of the variance (Table 6) and PC1 showed a strong positive correlation with TAC and TDS.

**Table 5:** PCA based on the common phytoconstituents detected from GC-MS analysis from the selected plant species

Principle Component Phytoconstituent	PC1	PC2	PC3	PC4	PC5	PC6	PC7	PC8
Pentasiloxane, dodecamethyl-	0.13	−0.05	−0.18	−0.33	0.33	−0.12	−0.52	−0.41
Glycerol, 3TMS derivative	−0.00	−0.04	0.53	−0.05	−0.11	0.08	−0.24	0.22
Silanol, trimethyl-, phosphate (3:1)	−0.21	0.03	0.10	0.17	0.37	−0.21	0.05	−0.16
Docosane	−0.10	−0.13	0.33	−0.22	−0.32	0.08	−0.27	−0.25
Nonadecane	−0.20	−0.17	−0.06	0.03	−0.02	0.10	−0.05	−0.20
Cyclononasiloxane, octadecamethyl-	0.10	−0.05	−0.13	−0.41	0.42	0.23	−0.29	0.52
Nonadecane, 9-methyl-	−0.12	0.28	−0.00	−0.10	0.06	−0.08	0.04	0.06
Pentadecane	−0.22	−0.18	−0.03	−0.01	0.02	0.01	−0.02	0.01
Palmitic Acid, TMS derivative	0.04	0.13	−0.03	0.48	−0.11	−0.02	−0.56	0.08
Heptadecane, 9-octyl-	−0.22	−0.18	−0.03	−0.01	0.02	0.01	−0.02	−0.06
Eicosane, 9-octyl-	−0.22	−0.18	−0.03	−0.01	0.02	0.01	−0.02	−0.06
Octadecane	−0.20	−0.09	−0.11	−0.01	−0.34	−0.40	−0.14	0.30
Eicosane, 1-iodo-	−0.17	0.05	0.12	0.30	0.42	−0.16	0.02	0.02
Dodecane, 2-methyl-	−0.12	0.28	−0.00	−0.10	0.06	−0.08	0.04	−0.02
Docosane, 1-iodo-	−0.09	0.27	−0.10	0.07	−0.08	0.28	−0.10	0.12
1-Monopalmitin, 2TMS derivative	−0.26	−0.05	−0.03	−0.06	0.04	−0.03	0.00	0.18
Eicosyl isopropyl ether	−0.22	−0.18	−0.03	−0.01	0.02	0.01	−0.02	0.01
Heptadecane, 3-methyl-	−0.26	−0.07	−0.02	−0.07	0.01	−0.05	−0.03	0.03
Octacosane, 1-iodo-	−0.15	0.24	0.06	−0.17	−0.06	−0.12	0.13	0.13
Glycerol monostearate, 2TMS derivative	−0.26	0.04	0.04	−0.13	0.03	0.01	0.03	0.11
Eicosane	−0.10	0.01	0.49	−0.11	0.06	0.26	0.03	−0.15
Heptacosane	−0.15	0.17	−0.13	0.24	−0.05	0.43	−0.17	0.02
Heneicosane	−0.09	0.25	0.19	−0.21	−0.03	0.09	0.05	−0.05
Hentriacontane	−0.24	−0.14	−0.03	−0.03	0.02	−0.01	−0.01	0.01
Octadecane, 3-methyl-	−0.24	0.10	−0.05	0.17	0.11	0.15	−0.07	−0.22
Hexacosane, 1-iodo-	−0.11	0.28	−0.05	−0.02	−0.01	0.10	−0.02	−0.21
Cyclobarbital	−0.22	−0.18	−0.03	−0.01	0.02	0.01	−0.02	0.01
Hexadecane, 7,9-dimethyl-	−0.12	0.28	−0.00	−0.10	0.06	−0.08	0.04	−0.06
Octacosane	−0.08	0.25	0.00	−0.10	−0.18	−0.43	−0.31	−0.04
Pentacosane	−0.25	0.09	−0.10	0.02	−0.03	0.19	−0.07	0.19
Methoxyacetic acid, 2-tridecyl ester	−0.12	0.28	−0.00	−0.10	0.06	−0.08	0.04	−0.02
Heptadecane, 2-methyl-	−0.22	−0.18	−0.03	−0.01	0.02	0.01	−0.02	0.01
Squalene	0.08	−0.03	0.43	0.24	0.29	−0.18	−0.08	0.20
Individual	0.43	0.31	0.10	0.08	0.04	0.03	0.02	0.00
Cumulative	0.43	0.73	0.83	0.91	0.95	0.98	1.00	1.00

**Table 6:** PCA analysis based on the combined measurements of the total phenolics, flavonoids, DPPH, TAC, and soil analysis

Principle Component Parameter	PC1	PC2	PC3	PC4	PC5	PC6	PC7	PC8
Total phenols	−1.07	0.06	0.04	0.00	0.01	−0.01	0.00	0.00
Flavonoids	−1.09	0.06	0.05	−0.00	0.00	−0.00	−0.00	0.00
TAC	4.89	−3.26	0.01	0.00	−0.00	0.00	0.00	−0.00
DPPH	−0.43	0.12	−0.42	0.00	−0.00	0.00	0.00	0.00
(EC)	−1.09	0.07	0.04	0.00	−0.00	0.00	0.00	−0.00
(TDS)	7.16	2.35	0.02	−0.00	0.00	−0.00	−0.00	0.00
Ca	−1.04	0.08	0.04	0.02	0.00	−0.00	−0.00	−0.00
Mg	−1.07	0.08	0.05	−0.01	−0.01	0.00	0.00	0.00
Na	−1.07	0.08	0.03	−0.01	0.01	0.00	−0.00	−0.00
K	−1.09	0.07	0.04	0.00	−0.00	0.00	0.00	−0.00
HCO_3_	−1.05	0.07	0.02	−0.01	0.01	0.00	0.00	−0.00
SO_4_	−1.04	0.09	0.06	0.01	−0.02	0.00	0.00	0.00
Cl	−1.07	0.07	0.03	0.01	0.01	0.00	−0.00	−0.00
pH	−0.94	0.06	−0.04	−0.02	−0.00	−0.01	−0.00	−0.00
Individual	0.57	0.23	0.1	0.07	0.02	0.01	0.00	0.00
Cumulative	0.57	0.8	0.9	0.97	0.99	1.00	1.00	1.00

**Fig. 9:**
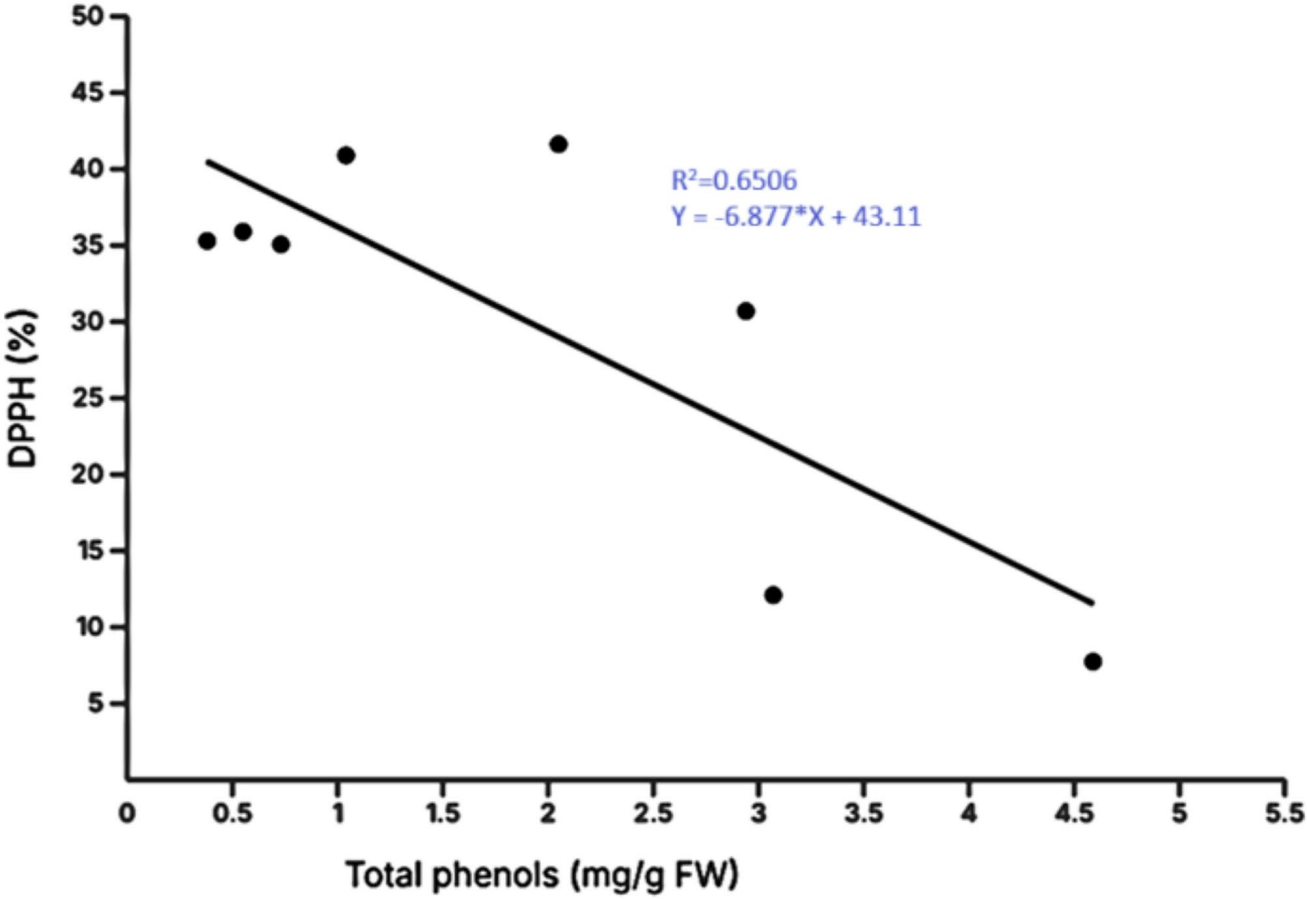
The relation between the DPPH (%) and Total Phenolics (mg/g FW) in the selected plant species

The analysis indicated a negative correlation between DPPH and Total phenolics. To assess this relationship, linear regression analysis was conducted, which was found to be statistically significant with a p-value of 0.0156 (p-value < 0.05) (Fig. 9). The coefficient of determination demonstrated a high degree of correlation, confirming the negative correlation between DPPH and Total phenolics.

**Fig. 10:**
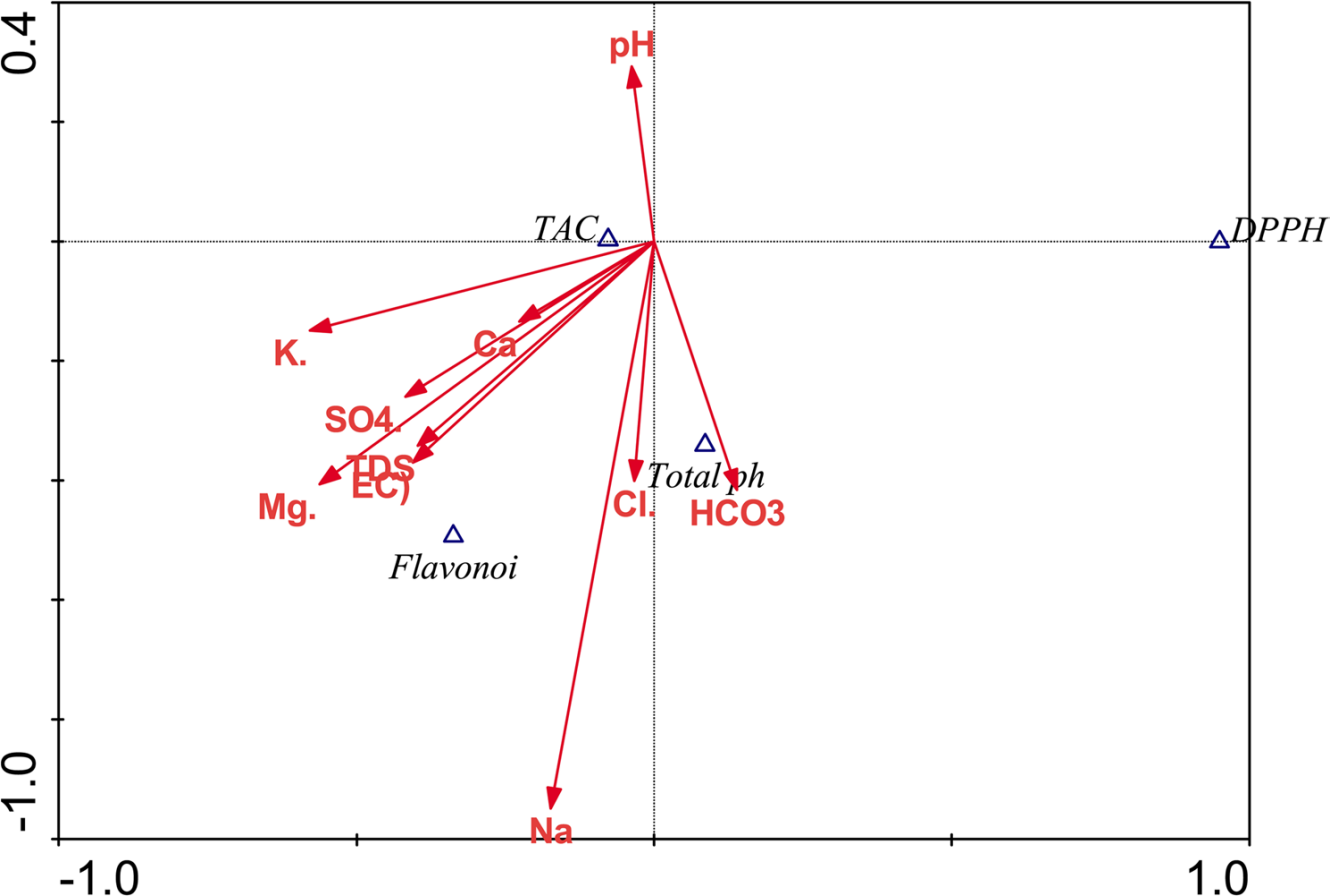
Biplot of Canonical Correspondence Analysis showing the relationships between plant analysis and soil chemistry

Canonical Correspondence Analysis (CCA) ordination program is an advanced statistical method widely used in ecology and environmental sciences. Canonical Correspondence Analysis (Fig. 10) showed the relation between soil parameters and the physiological aspects of plants and determined the effective parameters. The environmental variables were represented by arrows (↑) radiating from the center of the ordination plane. The direction and length of each arrow represent the ecological influence of that variable along the ordination axes. The longer the arrow, the stronger the relationship between the environmental variable and the vegetation distribution. The result of CCA analysis indicates the strong influence of soil chemistry and total phenolics, flavonoids and TAC. The CCA analysis showed a week relation between DPPH and soil chemistry.

### Characterization of the environmental impact on TCPs

**Fig. 11:**
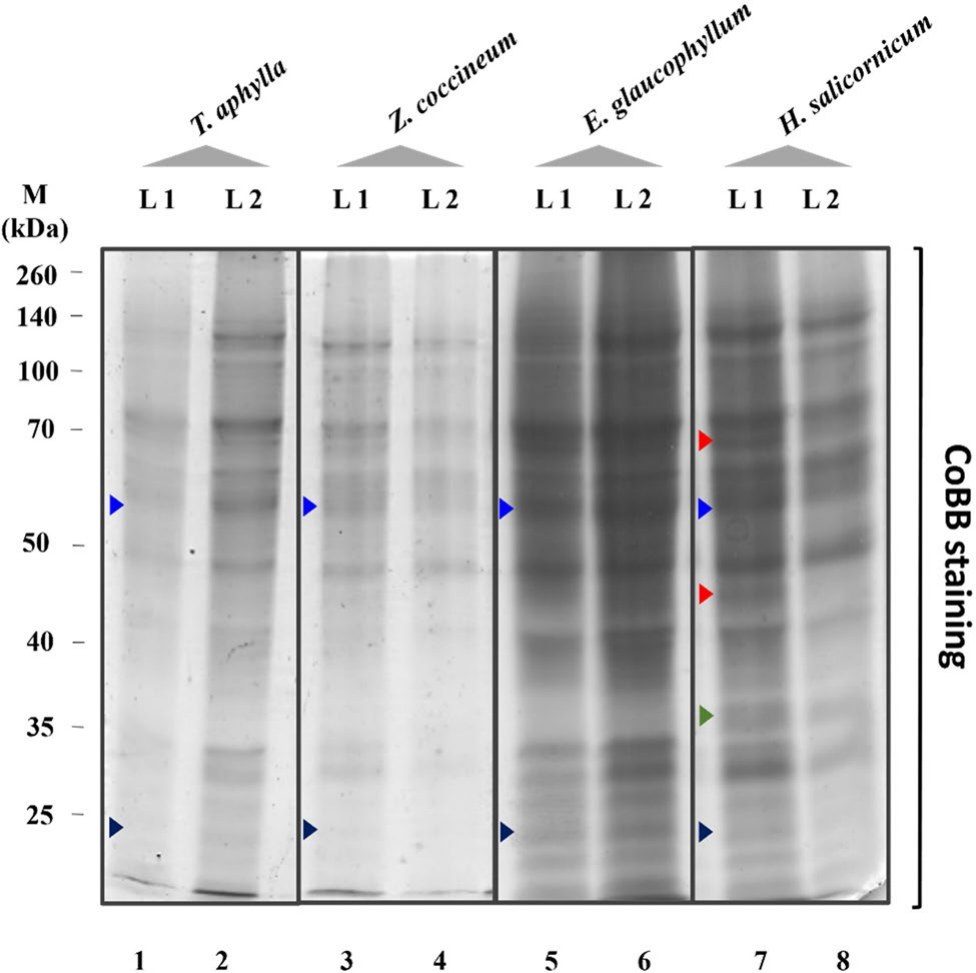
Electrophoretic banding patterns of extracted total cellular proteins (TCPs) of the four studied plant species from the two different locations (Al-Qalyubia (L1) and Al-Suez (L2) Governorates) using SDS-PAGE technique. Lane M: protein marker (Spectra™ Multicolor Broad Range Protein Ladder, Thermo Scientific™, Cat. number 26634), Lane 1: *T. aphylla* L1, Lane 2: *T. aphylla* L2, Lane 3: *Z. coccineum* L1, Lane 4: *Z. coccineum* L2, Lane 5: *E. glaucophyllum* L1, Lane 6: *E. glaucophyllum* L2, Lane 7: *H. salicornicum* L1, Lane 8: *H. salicornicum* L2. The numbers shown on the left-handed side of the figure refer to molecular weight standards in kDa (Spectra™ Multicolor Broad Range Protein Ladder). Red arrowheads refer to pronounced polypeptides detected in *H. salicornicum* L1, but barely or not detected in L2 sample. Light and dark blue arrowheads refer to approximate molecular weights of RuBisCO large (RbcLS) and small (RbcSS) subunits, respectively. Green arrowhead refers to a unique protein band running approximately at 30 kDa was detected in *H. salicornicum* (L1), but not detected in the rest of samples. Full-length uncropped and unprocessed gel accompanied this manuscript as Supplementary Information File and was presented in Supplementary Figure (Fig. S1)

TCPs were extracted from the leaves of eight studied taxa belonging to four plant species (listed in Table 1). Protein concentration of 100 µg TCPs (equivalent to total protein content) was unified between loaded samples and was separated via 12% SDS-PAGE technique (Fig. 11; Fig. S1). To affirm the homogeneity, consistency, and reproducibility of the protein profiles, TCPs were extracted from *Tamarix aphylla*, loaded, and fractionated using different ascending concentrations using SDS-PAGE technique (Fig. S1). Protein quantification for a specific protein band(s) was conducted using serially loaded ascending protein concentrations of bovine serum albumin (BSA) (Fig. S2). It was found that the SDS-PAGE technique resolved clear and distinct protein bands ranging generally between 15 and 130 kDa in all samples (Fig. 11). The results showed the detection of several pronounced protein bands running at approximately 130, 70, 50, and 45 kDa in all samples with various intensities (Fig. 11). At the species-specific level, several unique protein bands were detected in some taxa but were barely detected or absent on others. For example, in case of *H. salicornicum* two protein bands running at approximately 69 and 40 kDa were detected in *H. salicornicum* (L1) and absent in *H. salicornicum* (L2) (Fig. 11, Lanes 7–8, denoted by red arrowheads). In the same context, a unique protein band running at approximately 30 kDa was detected in *H. salicornicum* (L1) but not detected in the rest of samples (Fig. 11, Lanes 1–8, denoted by green arrowheads). Also, two major protein bands running at approximately 55 and 20 kDa were detected with notable fluctuations in all samples (Fig. 11, Lanes 1–8, denoted by light and dark blue arrowheads), respectively.

### Genetic relationships among the studied taxa as revealed by SDS-PAGE technique

**Fig. 12:**
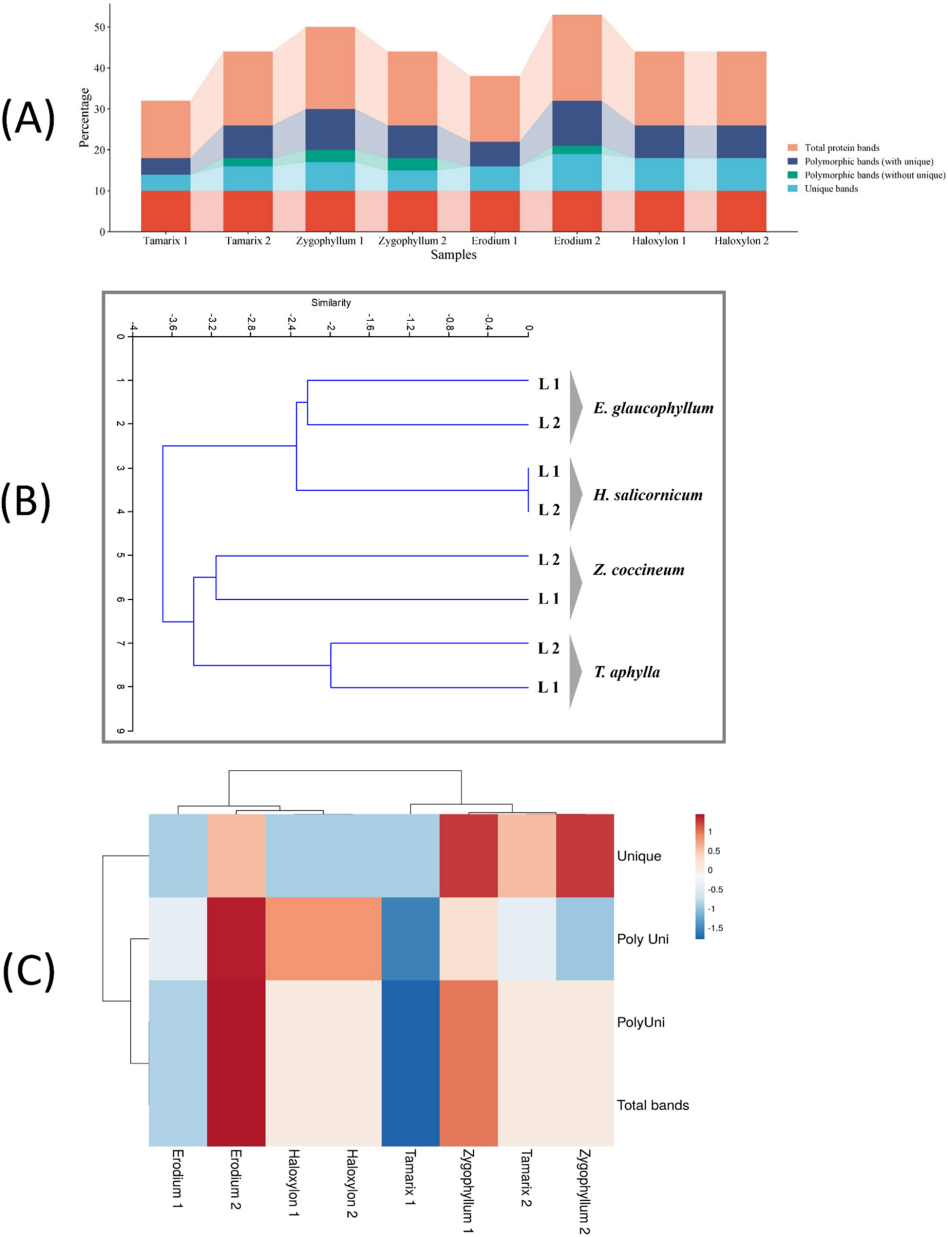
**A** A stacked bar chart showed total protein bands, polymorphic bands (including unique bands), polymorphic bands (without including unique bands), unique protein bands as revealed by the analysis of protein profiles using SDS-PAGE technique. **B** Cluster analysis was conducted based on the SDS-PAGE-separated total cellular proteins (TCPs) of the eight studied samples representing four different plant species. Latter dendrogram was constructed based on the scored data from the fractionated TCPs using ClustVis online free software tool (https://biit.cs.ut.ee/clustvis). **C **Multivariate Heatmap clustering was constructed to analyze the relationships among the studied plant taxa based on the employed SDS-PAGE fractionation of TCPs. The heatmap was generated using ClustVis online free software tool (https://biit.cs.ut.ee/clustvis)

Linear schematic representation of executed band scoring was shown (Fig. S3). The SDS-PAGE protein profiles and band scoring revealed a total of 38 protein bands with polymorphism percentage (P % = 73.68%) of 10 monomorphic and 28 polymorphic bands (Fig. 12a, Table S5). The Euclidean cluster analysis showed the differentiation of the eight samples into two main groups. In group I, the *T. aphylla* samples (L1 and L2) are grouped as one cluster, while *Z. coccineum* samples formed the second cluster. In group II, the samples *E. glaucophyllum* are recognized as a cluster, independent from the second one that included the *H. salicornicum* samples (Fig. 12b). Moreover, multivariate heatmap analysis was performed to construct a heatmap using the heatmap module of Viscluster software as shown downward in the Materials and Methods section. The conducted heatmap analysis separated the eight samples shown by the columns into two clusters that separated the samples of *T. aphylla* and *Z. coccineum* in a cluster, whereas *E. glaucophyllum* and *H. salicornicum* samples formed another cluster (Fig. 12c). The results of both the Euclidean distance tree (Fig. 12b) and the heatmap analysis (Fig. 12c) were in agreement and affirmed each other.

## Discussion

In general, environmental stress factors have a major impact on the production and storage of phytochemicals. Numerous elements, such as soil salinity, soil water status, light, and temperature, can have a significant impact on plant growth and its capacity to produce secondary metabolites. These factors can ultimately change entire phytochemical profiles, which in turn causes the accumulation of bioactive constituent [5, 22]. Variations in region may be linked to changes in total phenol and flavonoid levels as a result of different storage, processing, and environmental factors [23, 24].The changes in phenolics and flavonoids in plants collected from different geographical locations were previously recorded in many studies [23, 25–27]. According to Chaouqi [28],, a variety of environmental conditions have a significant impact on the accumulation of secondary metabolites in plants. Even when other parameters remain constant, a change in just one of these factors can modify the levels of secondary metabolites present.

The EC of a solvent is a measure of salt content as well as an indicator of electrolyte concentration. The EC of the nutritional solution influences the number of ions that plants may access through their root systems. Environmental factors influence the optimal EC, which varies depending on the plant [26, 29].

The EC and phenols measurements have a close correlation, the highest phenolics content found in *Tamarix aphylla* (from Q2) collected from the soil with the highest EC, matching results recorded by Aryal et al. [30] and Šamec et al. [31] and in contrast with the data of *H. salicornicum* (from Q2) has the lowest phenolics content. When plant parts are exposed to a variety of environmental challenges, flavonoids, an important component of plants, function as an additional antioxidant system [30, 32].

Biosynthesis of the secondary metabolites in plants is not only controlled genetically, but many biotic and abiotic stressors have a significant impact as well. Plants exhibit varied metabolic behaviors within distinct ecological niches to enhance their environmental adaptation [3]. The positive correlation between total phenolics and total flavonoids with the antioxidant capacity of plants as previously described [21, 23–35] agreed with the presented study. Hereby, it might be concluded that fluctuations in the soil parameters and growth conditions certainly affect the content in bioactive compounds. The synthesis of phytochemicals is directly impacted by the mineral concentration of the soil, including vital components like potassium, phosphorus, and nitrogen. Because plants have access to the building blocks they need to synthesize these molecules, studies have shown that nutrient-rich soils encourage larger concentrations of phytochemicals [36].

Changes in habitat have a major impact on the chemical composition, antioxidant, and antibacterial properties of plants gathered from both environments. The primary cause of this discrepancy is the increased concentration of polyphenolic chemicals [37] matching with our result as the difference in phenolic compound between the species from different location were significantly difference except for Erodium which has the lowest difference in soil composition between the two locations.

Negative correlation between total phenols and DPPH is previously recorded in many research as polyphenols are potent antioxidants. This means that as the concentration of total phenolics increases, the IC50 value (the concentration needed to scavenge 50% of DPPH radicals) decreases, indicating stronger antioxidant capacity [38, 39]. Variations in the relationship between bioactive chemicals and antioxidant activity may be due to a few variables, including the mechanisms of specific-action antioxidants in each test being compromised. These mechanisms include phenolic compounds’ capacity to absorb metal ions, inhibit lipid peroxidation, and reduce oxidative stress through electron or hydrogen transfer pathways [40].

Canonical Correspondence Analysis (CCA) showed strong relation between soil chemistry and total phenols, flavonoids and TAC and weak relation with DPPH. The same result was recorded by Farajpour [41]. High potassium showed a strong correlation with phenolic and flavonoids compounds [28]. Secondary metabolites pathways and their regulation are extremely sensitive to environmental changes [36].

The expression of different genes is significantly impacted by variations in precipitation, temperature, humidity, sunshine duration and intensity, solar radiation, altitude, and soil properties. These factors ultimately affect the synthesis of secondary metabolites, such as polyphenols, and the bioactive potential of plant species [42].

### Significant expression of cellular proteins under adverse ecological drivers

By attaining clear and focused protein profiles, it was prompted to detect and analyze the differential effect of surveyed ecological drivers and physiological responses on the expression levels of total cellular proteins. It was found that high quantitative variations, other than the qualitative ones, in the production of several proteins were revealed (Fig. 1, Fig S1). In this context, the rate of biosynthesis and degradation of cellular proteins, which is controlled by gene expression, is greatly affected by adverse abiotic conditions [42, 43]. Soil salinity determined by the total salts present in the liquid portion of the soil triggers deleterious effects not only on plant growth and crop production but also on the tilth and permeability of a soil [44]. In this context, pronounced fluctuations in protein profiles of salinity-stressed samples were detected through several polypeptides ranging from 63 to 180 kDa according to Hassanein et al. [45]. Khalifa et al. [46] investigated the fluctuation in the expression of a protein polypeptide running at approximately 60 kDA between up- and down regulation in the control and salt-stressed *pisum sativum*, respectively. The latter differentially expressed band was identified by nano HPLC-MS/MS as beta subunit of chloroplast ATP Synthase coupling factor 1 (CF1). Also, in the present study the protein profiles that belongs to the locations suffering high salt content were consistent with the findings of Manivannan et al. [47] who investigated the impact of salt stress on the cellular protein expression of *Capsicum annuum*. Furthermore, the protein profile was altered after in vitro selection of the NaCl-resistant mung bean and tomato as demonstrated by Hassan et al. [48]. The low expression levels of certain protein bands detected in case of *T. aphylla* and *Z. coccineum* collected from Q2, S1, or S3 locations might be attributed to the effect of high TDS content. As a result, oxidative stress was triggered and stimulated the production of reactive oxygen species. Consequently, down regulation of the expression of both cellular or organellar proteins was emerged [45, 46, 49, 50].

The presented manuscript demonstrated a protein band running at approximately 55 kDa with notable fluctuations among the studied taxa. This result was in accordance with the findings of Khalifa and El Ghandoor [51] and Khalifa et al. [46] who identified the prementioned protein band as Ribulose-1,5-bisphosphate carboxylase/oxygenase (RuBisCO) large subunit (RuBisCO_LS_). In this connection, Hassanein et al. [45, 50] referred to the same protein band extracted using the same procedure [52] and under the identical conditions by RuBisCO_LS_ protein. Therefore, it might be concluded that the latter protein band most likely identified as RuBisCO_LS_ in the current study. Moreover, the expression level of RuBisCO_LS_ band in the locations characterized by low TDS was much higher than those of high TDS content. This result agreed with Khalifa et al. [46] where salt-treated pea plants showed significant decrease in RuBisCO_LS_ biosynthesis under extreme NaCl conditions (300–400 mM). In the same context, RuBisCO_LS_ was down-regulated under salt stress conditions subjected to coriander samples [45]. The current study also investigated another protein polypeptide at approximately >20 kDa with varying expression levels among the studied taxa. The authors proposed that the latter polypeptide might be identified as RuBisCO small subunit (RuBisCO_SS_) supported by multiple perspectives. Whitney et al. [53] showed the migration of tobacco RuBisCO_SS_ at the same molecular weight (ca. >20 kDa, specifically 14.5 kDa) using the SDS-PAGE technique. Also, Bharti et al. [54] showed presence of RuBisCO two subunits; RuBisCO_LS_ and RuBisCO_SS_ detected at molecular weights of 56 and 14 kDa, respectively. However, these findings need to be further analyzed as future perspectives by immunoblotting techniques against specific antibodies of prementioned two proteins and/or distinct identification by nano HPLC-MS/MS. The rationale behind emphasizing the present study on the expression level of RuBisCO small and large subunits was because of the importance of RuBisCO regarded as the most abundant enzyme on earth responsible for the light-dependent reaction of photosynthesis [45, 50]. In higher plants, biosynthesis and assembly of active RuBisCO enzyme depends on the communication between the nucleus and chloroplast. The RuBisCO_SS_ subunits are encoded in the nucleus, synthesized in the cytosol, and transported to chloroplast. After importing into the chloroplast, RuBisCO_SS_ bind with RuBisCO_LS_ subunits which are encoded by the chloroplast genome [55]. As revealed by the cluster analysis grouped each studied plant species independently from the other taxa studied, it might be highlighted that these plant taxa followed the rule of geographical distribution. It could be concluded that monitored and studied ecological drivers and/or physiological responses showed consistent and conserved genetic background of studied taxa on the level of total cellular proteins.

This study has investigated the potential integration of ecological, physiological, and molecular analyses to evaluate and judge the effect of studied eco-physiological parameters to monitor and survey the climatic future conditions. Hereby, the information provided in this study may reflect initial insights and broaden the understanding of different sectors of beneficiaries including both academic and non-academic researchers, in sustaining the medicinal and economic significance of plant productivity according to Corwin and Yemoto [44].

## Conclusions

This study focused on differentiating and characterizing eight plant taxa collected from diverse locations by integrating ecological, physiological, and molecular analyses. Analysis of soil texture, soil parameters, metabolomics of phytoconstituents, and SDS-PAGE protein profiling indicated significant variations among plant taxa, providing valuable initial insights for identification and differentiation. This study shed the light on the correlation between plant total phenols, Flavonoids, TAC, DPPH, and soil analysis, showing a significant negative correlation between DPPH and soil EC. The clustering patterns of the studied species were related to some chemical properties in their soil. Recorded species with high difference in TDS and EC such as *H. salicornicum*, showed distinct separation, whereas those with low differences, such as *Z. coccienum*, clustered more closely. These results demonstrated differential adaptive strategies among species and may be considered as a guide for environmental conservation in stressed habitats. Also, analysis of the protein profiles reflected the impact of ecological drivers and physiological parameters on the expression level of total cellular proteins. The analysis investigated notable common characterized protein bands in all plant species, species-specific protein bands, and differential expression levels of other polypeptides linked to diverse climatic conditions in their destinations. From an ecological perspective, employing ecofriendly biostimulators (such as, humic-related substances and plant growth-promoting rhizobacteria (PGPRs)) is a safe discipline to enhance and improve the production of bioactive and healthy components. Future perspectives could also include marker-assisted selection by analyzing of DNA molecular markers, such as specific Short Tandem Repeats (STRs) and/or chloroplast DNA barcoding genes (e.g., *rpoC1*,* matK*, or *ndhF*) for a more refined and comprehensive analysis of genetic diversity.

## Materials and methods

### Study area and plant samples

For studying the difference in plant response to different environmental factors, four plant species were collected, each plant was collected from two different locations (Listed in Table 1): Location 1 Al-Qalyubia Governorate (from two sites) and location 2 Suez Governorate (from 3 different sites). Four perennial plant species were selected: *Tamarix aphylla* (L.) H.Karst. (family Tamaricaceae): the most well-known *Tamarix* species, reaching up to 18 m (60 feet) high. This plant is known with different names such as salt cedar, Athel tree, Athel tamarisk, and Athel pine. It is a treasure of various regions of the world including Central Africa, the Middle East, and Asia. *Zygophyllum coccineum* L. (Family Zygophyllaceae): The most common *Zygophyllum* species in Saudi Arabia and Egypt, which can be found in a variety of environments and soil types. It is a little perennial herb tolerant for saline soils and is widely distributed in the limestone wadies and plains of the Eastern (Arabian) desert. *Erodium glaucophyllum* (L.) L’Hér (Family Geraniaceae.): The perennial herbaceous plant is a hemicryptophyte species with flat leaves arranged in rosettes at the soil level. It is a familiar herb growing over large areas even where high levels of urban pollution exist, such as roadsides [56]. *Haloxylon salicornicum* (Moq.) Bunge ex Boiss. (FamilyAmaranthaceae): It is a much branched, perennial erect leafless shrub, woody at base. Stem and branches are pale yellow, jointed, joints produce into two short triangular points which take the place of leaves and are woolly within, flowers and fruits not observed. It is a fodder plant, mostly grazed by camels, and has high salt contents. All necessary agreements and permissions were obtained, adhering to the International Union for Conservation of Nature (IUCN) guidelines established at the 27th meeting of the IUCN Council, GLAND SWITZERLAND (1989). The Plant taxa were identified and verified by Dr. Amal Morsy, professor of plant ecology at Ain Shams University, Cairo, Egypt. Voucher specimens are deposited in the Department of Botany Herbarium, Ain Shams University (CAIA; http://sweetgum.nybg.org/science/ih/herbarium-details/?irn=123925). It is noteworthy that plant taxa investigated in this study were collected from the ordinations listed in Table 1.

### Climatic conditions

Meteorological variables were obtained from the NASA Prediction Of Worldwide Energy Resources (POWER) climate database, which provides satellite-based climatological data suitable for agro-environmental and ecological studies (Available from: https://power.larc.nasa.gov). Community level was selected as (Agroclimatology), temporal level was set as monthly and annual to the time extent (2013–2023), then the location was determined from the site’s coordinates, parameters in terms of precipitation (mm) (Fig. 1), temperature (°C) and relative humidity (%) (Fig. 2). The data were downloaded using the POWER Data Access Viewer in CSV format and subsequently processed in Microsoft Excel.

### Soil analysis

Soil samples from various stands throughout the research region were carefully taken at a single depth (020) using sealed tins. Each site had three samples taken. In March 2023, during the spring season, the samples were taken. The soil pebbles and pollutants were taken out, and the samples were dried in an oven to be used for additional study. Dry sieving, which separates soil fractions based on the Wentworth scale, was used to calculate particle size (%) [57, 58]. A portable pH meter (Model Ionlab pH level 1) was used to measure soil reaction (pH) in the 1:2. 5 soil solution [59]. The total dissolved salts (TDS) of the soil extracts were measured in mg/L using the method described by Jackson [60]. After the procedure outlined by Richards [59]and Rayan et al. [58], the soil extracts were analyzed for their anions and cations (Cl−, HCO3ˉ, SO42, Na+, Ca+², Mg+², and K+), and their values were given as meq/L. Using Collin’s Calcimeter [61], calcium carbonates was volumetrically measured.

### Plant analysis

#### Determination of total phenolic content

The total phenolic content was calculated using the procedures outlined by Makkar et al. [62]. and expressed in mg gallic acid equivalents/g dw.

#### Determination of flavonoids content

The aluminum chloride colorimetric technique [63] was used to identify flavonoids in the methanolic extract of the plant. A 0. 5 ml sample of the methanolic extract was diluted in 1. 5 ml of distilled water and then combined with 0. 5 ml of 10% aluminum chloride. The total flavonoids content was quantified using a standard curve of quercetin produced in 80% (v/v) methanol. Outcomes were reported as mg quercetin equivalent (QE) per gram of dry weight (dw).

#### Determination of total antioxidant capacity

The phosphomolybdenum technique, as described by Prieto et al. [64], was used to measure the overall antioxidant potential of the extract. The findings were presented as mg of gallic acid equivalents (GAE)/g dw.

#### Radical scavenging assay/activity (DPPH assay)

Following the methodology of Hatano et al. [65], 2, 2diphenyl1picrylhydrazyl (DPPH) radical scavenging activity was measured. Calculated as [(A0 A1)/A0] x 100, where A0 is the absorbance of the DPPH solution and A1 is the absorbance of the sample, the DPPH radical scavenging activity is presented. The standard curve was created using ascorbic acid; the DPPH scavenging activity was indicated as mM Ascorbic acid equivalents (ASAE)/g dw.

#### Gas chromatography (GC) analysis

1.5 g of each specimen was smashed into a powder using a ceramic mortar and pestle and allowed to dissolve in 30 mL of chloroform at room temperature for 48 h. The methanolic and chloroform extracts were then centrifuged for 15 min at room temperature (26–29 °C) at 3000 g (Universal 2 S, Hettich Zentrifugen, Tuttlingen, Germany) for gas chromatography-mass spectrometric (GC–MS) investigation using an Agilent 7890 A/5975 C GC–MSD equipment and a split (50:1) injection method. The GC included an Agilent 19091S433HP5MS capillary column (30. 00 m × 0. 25 mm inner diameter, 0. 25 μm phase thickness). Automated injection of 1 µL of sample, a runtime of 49 min, and a consistent carrier gas flow of 1. 5 mL/min of helium comprises the GC oven’s program. From 100 °C for four minutes to 300 °C at 4 °C/min, the temperature was raised then maintained isothermally at 240 °C for 10 min. The samples were inspected using full scan mode. The source temperature was 250 °C, the solvent delay was 5 min, and the electron ionization energy was 70 eV. The molecular weight, mass spectrum, and fragment ions from the mass spectrum of these compounds helped to identify them. These parameters were compared with those of reference compounds retrieved from the National Institute of Standards and Technology 2011 database and integrated into the computer system of the instrument. Hereby, the identification was performed by matching the obtained spectra with the NIST 2011 library using a similarity index ≥ 90%. No authentic standards or retention index (RI) calculations were applied. Therefore, the identifications were putative. Polyhydroxylated alkaloids, for example, are among the substances that are difficult to vaporize. Silylation or replacing the hydroxyl group with other chemical groups such trimethylsilyl groups before injection onto the GCMS, may improve their ability to vaporize and has been carried out using N, O-bis(trimethylsilyl)acetamide (BSA; 100 µL, 70 °C, 2 h) before injection for non-volatile compounds.

#### Extraction of total cellular proteins (TCPs)

Extraction of TCPs were performed from the leaves of the eight samples (listed in Table 1). From the leaves of three biological and three technical replicates, the procedure of protein extraction from the eight studied taxa was executed. Each biological replicate included the collection of the leaves of ten plants as previously described by Hassanein et al. [45, 50]. Physical cellular disruption was performed using high throughput TissueLyser II equipment (Qiagen, Cay. No. 85300) for three times/30 seconds each for tissue aliquots (250 mg). Immediately, TCPs were isolated in 100 µl extraction buffer of 2% w/v SDS, 4% β-mercaptoethanol, 50 mM EDTA, 40% Glycerol, and 100 mM Tris-HCl (pH 8). The extraction buffer was supplemented with bromophenol blue dye (0.001%), 1x protease inhibitors cocktail (Roche, Penzberg, Germany), and 0.1 mM phenylmethylsulfonyl fluroide (PMSF). The cellular homogenate was thoroughly mixed till a complete homogeneous lysate was obtained via the mortar and pestle. Subsequently, the tissue lysate was vortexed for five minutes, incubated at 95 °C for three minutes (Eppendorf ^TM^ Thermomixer ™), and finally centrifuged (MICRO 22- MICRO Centrifuge, Hettich, Germany) for 15 min at 20.000 xg. Then, the extracted TCPs were stored as aliquots (25 µl) at −80 °C freezer for subsequent analysis by the SDS-PAGE technique. Quantification of protein concentration (equivalent to total protein content) was measured using protein assay dye reagent (BioRad, cat No. #5000006) as demonstrated by Hassanein et al. [45, 50].

#### SDS-PAGE technique and cluster analysis

Previously extracted TCPs were subjected to either preparatory and/or analytical one-dimensional 12% SDS-PAGE procedure as previously described [50, 52]. Electrophoresis process was executed using omniPAGE Mini Wide vertical slap (Cleaver scientific. Ltd, UK) following the manufacturer recommendations. The pre-stained color-coded protein marker (Spectra™ Multicolor Broad Range Protein Ladder, Thermo Scientific™, Cat. number 26634) was loaded. Gel images were documented using Bio-Rad gel documentation system (Gel DocTM EZ system and enabled Image Lab TM software). Band Scoring and the binary matrix were generated using the separated protein patterns (as revealed by SDS-PAGE technique) to calculate the genetic similarity matrix coefficient.

### Statistical analysis

In case of soil analysis, three soil samples have been collected from each location for analysis. Significant differences were calculated after the performance of one-way ANOVA analysis of variance and post-hoc (Duncan) were proceeded using IBM Corp (2017). IBM SPSS Statistics for Windows, Version 25.0. Armonk, NY: IBM Corp. In case of the measurements of plant physiological parameters, three biological samples were collected from each selected plant and the experiment were measured in triplicates. Significant differences were calculated after the performance of one-way ANOVA analysis of variance and post hoc LSD for plant analysis were proceeded using IBM Corp (2017). IBM SPSS Statistics for Windows, Version 25.0. Armonk, NY: IBM Corp. To conduct the correlation analysis, Pearson correlation was generated using IBM Corp (2017). IBM SPSS Statistics for Windows, Version 25.0. Armonk, NY: IBM Corp. for linear regression Microsoft Excell program was used. Multivariable heatmap correlation analysis was carried out online with the free online site (https://biit.cs.ut.ee/clustvis/). The CAP program version 1. 3. 1 was used to carry out the PCA correlation [66]. Ballon’s size and colors were used to show the values strength from SRplot free online platform for data visualization and graphing (https://bioinformatics.com) [67]. Canonical Correspondence Analysis (CCA) was applied for analyzing the physiological parameters measured of plants and soil analysis. The ordination axes represent gradients of change in community composition. CCA produces a biplot graphical presentation of the interrelationships between plant analysis groups and environmental variables in one graph, along the four axes of the ordination plane. CCA was performed using the CANOCO for windows program, version 4.5.2 [68]. In case of protein profiling, three biological replicates and three technical replicates were used. TCPs were isolated from the three technical replicates independently. Extracted TCPs from the three technical replicates were pooled in one sample for further use in SDS-PAGE technique as described by Hassanein et al. [50]. The SDS-PAGE binary data (scoring “1” for presence and “0” for absence) was used to construct Euclidean distance tree. Clustering analysis was performed to detect the relatedness among the samples studied, as indicated by their protein patterns. By using PAST software, ver. 4.02 and By employing the unweighted pair group method with arithmetic mean (UPGMA) algorithm, a distance tree was constructed following Hammer et al. [69]. To assure the results of the Euclidean distance tree, multivariate heatmap-based analysis was performed using ClustVis software (https://biit.cs.ut.ee/clustvis); online free web tool for visualizing clustering of multivariate data as previously described by Metsalu and Vilo [70].

## Supplementary Information

Supplementary Material 1.

## Data Availability

All datasets generated and/or analyzed during this study were completely included within the article and its supplementary information file.
