# Supplementary material for: Eco-physiological adaptations, metabolomic profiles and genetic diversity across varied habitats in four medicinal plant species
Source: BMC Plant Biol. 2025 Nov 14;25:1566. doi: 10.1186/s12870-025-07521-7 (PMC12616984; doi:10.1186/s12870-025-07521-7)
Supplement: Supplementary file 1 — Supplementary Material 1. [file 12870_2025_7521_MOESM1_ESM.docx]

**Supplementary Tables and Figures**

**Eco-Physiological Adaptations, Metabolomic Profiles and Genetic Diversity Across Varied Habitats in Four Medicinal Plant Species**

**Gamal E.A., Morsy A.A., Maher M.** **Shehata, Mohamed Ibrahim*, Saleh H.A.**

Department of Botany, Faculty of Science, Ain Shams University, 11355, Cairo, Egypt

*Corresponding author: [m.shehata@sci.asu.edu.eg](mailto:m.shehata@sci.asu.edu.eg) (M.I.) ORCID no. 0000-0002-5401-5115

**Supplementary Table S1** Phytoconstituent identified by GC-MS analysis of *T. aphylla* chloroform extract. R.T.=Retention Time; MF=Molecular formula; M. wt. = Molecular weight.

| No | RT | Library/ID | MF | M.wt | *Tamarix* 1 | *Tamarix* 2 |
| --- | --- | --- | --- | --- | --- | --- |
| 1 | 8.9451 | Pentasiloxane, dodecamethyl- | C_12_H_36_O_4_Si_5_ | 384.8393 | 0.14 | 0 |
| 2 | 8.9226 | Silanol, trimethyl-, carbonate (2:1) | C_7_H_18_O_3_Si_2_ | 206.39 | 0 | 0.3156 |
| 3 | 9.0657 | 2-Ketoglutaric acid, 2-(ethoxyimino)-, bis(trimethylsilyl) ester | C_12_H_25_NO_5_Si_2_ | 319.5016 | 0 | 0.29 |
| 4 | 9.2599 | 6-Chlorohexanoic acid, TMS derivative | C_6_H_11_ClO_2_ | 150.60 | 0.0674 | 0 |
| 5 | 9.3972 | Trisiloxane, 1,1,1,5,5,5-hexamethyl-3,3-bis[(trimethylsilyl)oxy]- | C_12_H_36_O_4_Si_5_ | 384.8393 | 0.1624 | 0 |
| 6 | 9.7348 | Glycerol, 3TMS derivative | C_12_H_32_O_3_Si_3_ | 308.6372 | 0.8597 | 1.3257 |
| 7 | 9.998 | Silanol, trimethyl-, phosphate (3:1) | C_9_H_27_O_4_PSi_3_ | 314.5385 | 1.9676 | 1.7884 |
| 8 | 10.3131 | Pyrimidine-4-ol, 2,6-diamino-5-benzylidenamino- | C_4_H_5_N_5_O_2_ | 155.12 | 0 | 0.5459 |
| 9 | 10.4847 | Anthracene, 9-methyl- | C_15_H_12_ | 192.2558 | 0 | 1.5308 |
| 10 | 10.5759 | [1,1'-Biphenyl]-4-carbonitrile, 4'-propyl- | C_16_H_15_N | 221.297 | 0.8269 | 0 |
| 11 | 10.7018 | 4,7-Dimethoxyindan-1-one | C_11_H_12_O | 160.21 | 0.4826 | 0 |
| 12 | 10.8048 | 4H-1,2,4-triazol-3-amine, 5-(phenylthio)- | C_3_H_6_N_4_S | 130.17 | 0.6946 | 0 |
| 13 | 10.8051 | 1H-Indene, 2-phenyl- | C_15_H_12_ | 192.2558 | 0 | 0.4933 |
| 14 | 10.9081 | 3-Fluoro-5-(trifluoromethyl)benzaldehyde | F_3_CC_6_H_3_(F)CHO | 192.11 | 0 | 0.6469 |
| 15 | 11.2454 | 2-Bromo dodecane | C_12_H_25_Br | 249.231 | 2.4939 | 0 |
| 16 | 11.5772 | 2,6-Bis(tert-butyl) phenol, TMS derivative | C_14_H_22_O | 206.32 | 4.5872 | 3.8344 |
| 17 | 12.0693 | Docosane | C_22_H_46_ | 310.6 | 0.4965 | 2.7888 |
| 18 | 12.4931 | Nonadecane | C_19_H_40_ | 268.5 | 0 | 1.7869 |
| 19 | 13.1226 | Cyclononasiloxane, octadecamethyl- | C_18_H_54_O_9_Si_9_ | 667.3855 | 0 | 0.1706 |
| 20 | 13.4827 | Nonadecane, 9-methyl- | C_20_H_42_ | 282.5475 | 1.0873 | 0 |
| 21 | 14.3928 | Octadecane, 3-ethyl-5-(2-ethylbutyl)- | C_26_H_54_ | 366.7070 | 0 | 0.3243 |
| 22 | 14.5016 | Pentadecane | C_15_H_32_ | 212.4146 | 0 | 0.751 |
| 23 | 14.5069 | 2-methyloctacosane | C_29_H_60_ | 408.7867 | 0.2946 | 0 |
| 24 | 15.0104 | 2-Quinolinecarboxylic acid, 6-bromo-1,4-dihydro-4-oxo-, ethyl ester | C_12_H_10_BrNO_3_ | 296.12 | 0.0808 | 0 |
| 25 | 15.3709 | Palmitic Acid, TMS derivative | C_19_H_40_O_2_Si | 328.6052 | 2.9333 |  |
| 26 | 15.3599 | Heptadecane, 9-octyl- | C_25_H_52_ | 352.6804 | 0 | 1.6705 |
| 27 | 15.8348 | Eicosane, 9-octyl- | C_28_H_58_ | 394.7601 | 0 | 1.1351 |
| 28 | 16.0862 | Octadecane | C_18_H_38_ | 254.4943 | 1.2247 | 3.3714 |
| 29 | 16.9616 | Coronene, methyl- | C_25_H_14_ | 314.4 | 1.5786 | 0 |
| 30 | 17.2138 | 6,6-Diethylhoctadecane | C_20_H_42_ | 282.5475 | 0 | 1.8286 |
| 31 | 17.2306 | Stearic acid, TMS derivative | C_21_H_44_O_2_Si | 356.6584 | 2.386 | 0 |
| 32 | 17.6944 | Hexadecane, 2,6,10,14-tetramethyl- | C_20_H_42_ | 282.5475 | 1.2558 | 0.3254 |
| 33 | 18.003 | Eicosane, 1-iodo- | C_20_H_41_I | 408.444 | 1.5671 | 1.674 |
| 34 | 18.3749 | Tetracosane, 1-iodo- | C_24_H_49_I | 464.5503 | 1.6146 | 0 |
| 35 | 19.2222 | 4-Methylthio-N-phenyl-1,2-carbazoledicarboximide | C_21_H_14_N_2_O_2_S | 358.4 | 1.1399 | 0 |
| 36 | 19.8054 | Dodecane, 2-methyl- | C_13_H_28_ | 184.3614 | 4.1513 | 0 |
| 37 | 20.0057 | Docosane, 1-iodo- | C_22_H_45_I | 436.4972 | 1.2679 | 0 |
| 38 | 20.1488 | 1-Monopalmitin, 2TMS derivative | C_25_H_54_O_4_Si_2_ | 474.8649 | 3.5568 | 7.7247 |
| 39 | 20.6527 | Eicosyl isopropyl ether | C_23_H_48_O | 340.6266 | 0 | 1.2089 |
| 40 | 20.996 | Triacontane, 1-iodo- | C_30_H_61_I | 548.7098 | 0 | 1.7127 |
| 41 | 21.0185 | Heptadecane, 3-methyl- | C_18_H_38_ | 254.4943 | 2.2687 | 5.6963 |
| 42 | 21.2878 | 2-Monostearin, 2TMS derivative | C_27_H_58_O_4_Si_2_ | 502.9180 | 0 | 9.4215 |
| 43 | 21.3103 | Octacosane, 1-iodo- | C_28_H_57_I | 520.6567 | 7.0251 | 1.4969 |
| 44 | 21.5396 | Glycerol monostearate, 2TMS derivative | C_27_H_58_O_4_Si_2_ | 502.9180 | 6.1408 | 7.1881 |
| 45 | 22.5062 | Eicosane | C_20_H_42_ | 282.54 | 8.5437 | 9.0234 |
| 46 | 22.7065 | Heptacosane | C_27_H_56_ | 380.7335 | 2.9649 | 1.4795 |
| 47 | 22.9529 | Triacontane | C_30_H_62_ | 422.8133 | 0 | 2.5199 |
| 48 | 22.9811 | Heneicosane | C_21_H_44_ | 296.5741 | 9.7626 | 0 |
| 49 | 23.1932 | Hentriacontane | C_31_H_64_ | 436.8 | 0.4703 | 3.3291 |
| 50 | 23.5419 | Octadecane, 3-methyl- | C_19_H_40_ | 268.5209 | 5.7029 | 5.233 |
| 51 | 24.0225 | Hexacosane, 1-iodo- | C_26_H_53_I | 492.6035 | 9.2725 | 0 |
| 52 | 24.4349 | Cyclobarbital | C_12_H_16_N_2_O_3_ | 236.27 | 0 | 5.0666 |
| 53 | 24.4746 | Hexadecane, 7,9-dimethyl- | C_18_H_38_ | 254.4943 | 1.6605 | 0 |
| 54 | 24.8812 | 11-Methylpentacosane | C_26_H_54_ | 366.7070 | 0 | 2.4 |
| 55 | 25.104 | Octacosane | C_28_H_58_ | 394.7601 | 1.9827 | 0 |
| 56 | 25.4702 | Pentacosane | C_25_H_52_ | 352.6804 | 6.5909 | 6.4789 |
| 57 | 25.8993 | Methoxyacetic acid, 2-tridecyl ester | C_16_H_32_O_3_ | 272.42 | 1.4704 | 0 |
| 58 | 27.7079 | Heptadecane, 2-methyl- | C_18_H_38_ | 254.4943 | 0 | 0.2425 |

**Supplementary Table S2** Phytoconstituent identified by GC-MS analysis of *Z. coccineum* chloroform extract. R.T.=Retention Time; MF=Molecular formula; M. wt. = Molecular weight.

| PK | RT | Library/ID | MF | M.wt | *Zygophyllum* 1 | *Zygophyllum* 2 |
| --- | --- | --- | --- | --- | --- | --- |
| 1 | 8.8252 | Acetic acid, bis[(trimethylsilyl)oxyl]-, trimethylsilyl ester | C_11_H_29_O_5_PSi_3_ | 356.57 | 6.0083 | 0 |
| 2 | 9.0713 | Boric acid, 3TMS derivative | C_9_H_27_BO_3_Si_3_ | 278.376 | 3.5466 | 0 |
| 3 | 9.2715 | Pentasiloxane, dodecamethyl- | C_12_H_36_O_4_Si_5_ | 384.8393 | 5.3781 | 2.2671 |
| 4 | 9.2827 | Trisiloxane, 1,1,1,5,5,5-hexamethyl-3,3-bis[(trimethylsilyl)oxy]- | C_12_H_36_O_4_Si_5_ | 384.8393 | 0 | 0.655 |
| 5 | 9.7637 | Glycerol, 3TMS derivative | C_12_H_32_O_3_Si_3_ | 308.6372 | 1.6233 | 3.7295 |
| 6 | 10.1123 | Isoborneol, pentamethyldisilanyl ether | [C_15_H_32_OSi_2_](https://pubchem.ncbi.nlm.nih.gov/#query=C15H32OSi2) | 284.58 | 0 | 2.8534 |
| 7 | 10.25 | Phenanthrene, 2-methyl- | C_15_H_12_ | 192.2558 | 20.4892 | 0 |
| 8 | 10.63388 | Modephene | C_15_H_24_ | 204.3511 | 0 | 4.1976 |
| 9 | 10.7818 | 5-Ethynyl-7-methoxy-2H-1,3-benzodioxol-4-ol | C_16_H_12_O_5_ | 284.26 | 0 | 6.1778 |
| 10 | 11.2396 | Dimethylthexylsilyl chloride | C_8_H_19_ClSi | 178.77 | 0 | 0.848 |
| 11 | 10.8451 | Phenanthrene, 4-methyl- | C_15_H_12_ | 192.2558 | 2.1453 | 6.0831 |
| 12 | 11.217 | Hexadecane | C_16_H_34_ | 226.44 | 4.4678 | 0 |
| 13 | 11.5718 | 2-Hydroxybenzimidazole, N, O-bis(trimethylsilyl)- | C_13_H_22_N_2_OSi_2_ | 278.4976 | 2.379 | 2.0436 |
| 14 | 11.9323 | cis-1,2-Bis(diphenylphosphino)ethylene | C_26_H_22_P_2_ | 396.4 | 1.4213 | 0 |
| 15 | 12.4759 | Octadecane | C_18_H_38_ | 254.4943 | 2.1557 | 0 |
| 16 | 12.7505 | Octacosane, 1-iodo- | C_28_H_57_I | 520.6567 | 0.911 | 1.4447 |
| 17 | 13.7805 | Nonadecane, 9-methyl- | C_20_H_42_ | 282.5475 | 1.1732 | 0 |
| 18 | 14.0666 | 2-Methylhexacosane | C_27_H_56_ | 380.7335 | 0.7666 | 0 |
| 19 | 14.1063 | Pentacosane | C_25_H_52_ | 352.6804 | 0 | 0.867 |
| 20 | 14.4496 | Hexacosyl propyl ether | C_29_H_60_O | 424.7861 | 0 | 1.0459 |
| 21 | 14.4728 | Hentriacontane | C_21_H_44_ | 296.5741 | 0.5119 | 0 |
| 22 | 14.833 | Cedranoxide, 8,14- | C_15_H_24_O | 220.3505 | 0 | 0.8333 |
| 23 | 14.9932 | 4-Hydroxybenzaldoxime, 2TMS derivative | C_13_H_23_NO_2_Si_2_ | 281.4982 | 0 | 0.2721 |
| 24 | 14.9993 | Hexadecanoic acid, ethyl ester | C_18_H_36_O_2_ | 284.4772 | 7.6895 | 4.0426 |
| 25 | 15.354 | Palmitic Acid, TMS derivative | C_19_H_40_O_2_Si | 328.6052 | 3.8331 | 0 |
| 26 | 16.0578 | Heptadecane, 9-octyl- | C_25_H_52_ | 352.6804 | 1.6111 | 0.45522 |
| 27 | 16.3668 | Disulfide, di-tert-dodecyl | C_24_H_50_S_2_ | 402.8 | 0.5347 | 0 |
| 28 | 16.4523 | Hexasiloxane, tetradecamethyl | C_14_H_42_O_5_Si_6_ | 458.9933 | 0 | 0.9896 |
| 29 | 16.4641 | Dotriacontane, 1-iodo- | C_32_H_65_I | 576.7630 | 0.6477 | 0 |
| 30 | 16.8818 | Octadecanoic acid, ethyl ester | C_20_H_40_O_2_ | 312.5304 | 4.4855 | 0 |
| 31 | 17.2019 | Cyclononasiloxane, octadecamethyl- | C_18_H_54_O_9_Si_9_ | 667.3855 | 0 | 2.2814 |
| 32 | 17.8027 | Heptasiloxane, hexadecamethyl- | C_16_H_48_O_6_Si_7_ | 533.1472 | 0 | 0.9356 |
| 33 | 17.9918 | Heptadecane, 3-methyl- | C_18_H_38_ | 254.4943 | 0.3436 | 0.2421 |
| 34 | 19.0329 | Mercaptoacetic acid, 2TMS derivative | C_13_H_22_O_2_SSi_2_ | 298.549 | 0 | 0.6458 |
| 35 | 19.3594 | Hexadecane, 2,6,10,14-tetramethyl- | C_20_H_42_ | 282.5475 | 0.3355 | 0 |
| 36 | 19.3762 | 2-Propenoic acid, 3-(4-methoxyphenyl)-, 2-ethylhexyl ester | C_18_H_26_O_3_ | 290.3972 | 0 | 1.0351 |
| 37 | 19.5536 | 1,1':3',1''-Terphenyl, 4,4''-diethyl-5'-(4-ethylphenyl)- | C_24_H_22_O_4_ | 374.4 | 0 | 2.494 |
| 38 | 19.7256 | Carbonic acid, eicosyl vinyl ester | C_23_H_44_O_3_ | 368.5937 | 0.833 | 0 |
| 39 | 19.731 | Decane, 3,8-dimethyl- | C_12_H_26_ | 170.3348 | 0 | 1.1572 |
| 40 | 20.0346 | 9-(2',2'-Dimethylpropanoilhydrazono)-3,6-dichloro-2,7-bis-[2-(diethylamino)- | C_30_H_42_C_l2_N_4_O_3_ | 577.6 | 1.2173 | 0 |
| 41 | 20.1258 | Piperidine, 1-(5-trifluoromethyl-2-pyridyl)-4-(1H-pyrrol-1-yl)- | C_15_H_16_F_3_N_3_ | 295.30 | 0 | 1.7254 |
| 42 | 20.2463 | Docosanoic acid, ethyl ester | C_24_H_48_O_2_ | 368.6367 | 0.4654 | 0 |
| 43 | 20.6465 | 1,1,1,5,7,7,7-Heptamethyl-3,3-bis(trimethylsiloxy)tetrasiloxane | C_16_H_48_O_6_Si_7_ | 533.1472 | 0 | 0.2539 |
| 44 | 20.8586 | Heneicosane | C_21_H_44_ | 296.5741 | 0.4131 | 4.5228 |
| 45 | 21.0016 | Nonadecane | C_19_H_40_ | 268.5 | 1.77 | 0 |
| 46 | 21.1904 | Heptacosane, 1-chloro- | C_27_H_55_Cl | 415.179 | 0.4727 | 0 |
| 47 | 21.5162 | Glycerol monostearate, 2TMS derivative | C_27_H_58_O_4_Si_2_ | 502.9180 | 0 | 1.3793 |
| 48 | 21.5337 | Methoxyacetic acid, 2-pentadecyl ester | C_18_H_36_O_3_ | 300.5 | 0.4418 | 0 |
| 49 | 21.8656 | Squalene | C_30_H_50_ | 410.7 | 0.4169 | 0.8923 |
| 50 | 22.6781 | Docosane | C_22_H_46_ | 310.6 | 1.7952 | 3.2628 |
| 51 | 22.9013 | Hexacosane | C_21_H_44_ | 296.5741 | 1.3312 | 0 |
| 52 | 23.3187 | (-)-cis-3,4-Dimethyl-2-phenyltetrahydro-1,4-thiazine | C12H17NS | 207.34 | 0 | 1.5893 |
| 53 | 23.525 | Triacontane, 1-bromo- | C_30_H_61_Br | 501.709 | 5.3639 | 0 |
| 54 | 23.7825 | 1-Octacosanol, TBDMS derivative | C_34_H_72_OSi | 525.0204 | 1.6219 | 0 |
| 55 | 23.9942 | Z,Z-6,26-Pentatriacontadien-2-one | C_35_H_72_ | 492.9462 | 1.7131 | 0 |
| 56 | 24.1372 | Eicosane | C_20_H_42_ | 282.54 | 1.4410 | 16.5795 |
| 57 | 24.4058 | 1-(3-Methylbutyl)-2,3,6-trimethylbenzene | C_14_H_22_ | 190.3245 | 0 | 9.2943 |
| 58 | 24.5889 | Heneicosane, 3-methyl- | C_22_H_46_ | 310.6006 | 0 | 2.162 |
| 59 | 24.841 | 1-Bromo-11-iodoundecane | C_11_H_22_BrI | 361.10 | 1.6606 | 0 |
| 60 | 25.0238 | Tetratriacontane | C_34_H_70_ | 478.9196 | 0 | 3.4526 |
| 61 | 25.0413 | Stigmasterol, TMS derivative | C_32_H_56_OSi | 484.8719 | 0.8619 | 0 |
| 62 | 25.4472 | Eicosyl isopropyl ether | C_23_H_48_O | 340.6266 | 0 | 2.781 |
| 63 | 25.4533 | Stigmast-5-ene, 3.beta.-(trimethylsiloxy)-, (24S)- | C_32_H_58_OSi | 486.8878 | 3.2335 | 0 |
| 64 | 25.6192 | Octacosane | C_28_H_58_ | 394.7601 | 1.1474 | 0 |
| 65 | 25.9969 | 2,6,10,14-Tetramethyl-7-(3-methylpent-4-enylidene) pentadecane | C_25_H_48_ | 348.6486 | 0.4629 | 0 |
| 66 | 27.1295 | Nonacos-1-ene | C_29_H_52_ | 400.7232 | 0 | 0.523 |
| 67 | 27.2271 | Hexatriacontyl trifluoroacetate | C_38_H_73_F_3_O_2_ | 618.9802 | 0.8705 | 0 |

**Supplementary Table S3** Phytoconstituent identified by GC-MS analysis of *E. glaucophyllum* chloroform extract. R.T.=Retention Time; MF=Molecular formula; M. wt. =Molecular weight.

| PK | RT | Library/ID | MF | M.wt | *Erodium* 1 | *Erodium* 2 |
| --- | --- | --- | --- | --- | --- | --- |
| 1 | 8.8306 | Cyclopentasiloxane, decamethyl- | C_10_H_30_O_5_Si_5_ | 370.7697 | 0 | 7.717 |
| 2 | 9.4658 | Pentasiloxane, dodecamethyl- | C_12_H_36_O_4_Si_5_ | 384.8393 | 0.6727 | 13.7984 |
| 3 | 9.8377 | L-(+)-Threose, tris(trimethylsilyl) ether, ethyloxime (isomer 1) | C_14_H_35_NO_4_Si_3_ | 365.6885 | 0 | 1.2744 |
| 5 | 10.0896 | 9bH-1,3,4,6,7,9,9b-Heptaazaphenalene-2,5,8-triol | C_6_H_3_N_7_O_3_ | 221.14 | 0.1696 | 0 |
| 6 | 10.3698 | 4-Methylesculetin | C_10_H_8_O_4_ | 192.17 | 0 | 7.8681 |
| 7 | 10.8047 | Anthracene, 1-methyl- | C_15_H_12_ | 192.255 | 0 | 4.5139 |
| 8 | 10.8049 | 1,3-Benzodioxole, 4-methoxy-6-(2-propenyl)- | C_11_H_12_O_3_ | 192.21 | 0.9749 | 0 |
| 9 | 11.1425 | .alpha.-Damascone | C_13_H_20_O | 192.30 | 0.5696 | 0 |
| 10 | 11.2339 | 4-Fluoro-2-(trifluoromethyl)benzaldehyde | C_8_H_4_F_4_O | 192.11 | 0 | 4.0892 |
| 11 | 11.2455 | Octadecane, 1-iodo- | C_28_H_57_I | 520.6567 | 0.9835 | 0 |
| 12 | 11.5201 | 2,4-Di-tert-butylphenol | C_14_H_22_O | 206.32 | 6.1455 | 0 |
| 13 | 12.1324 | 2,5-Dihydroxybenzoic acid, 3TMS derivative | C_16_H_30_O_4_Si_3_ | 370.66 | 0.878 | 0 |
| 14 | 11.5829 | Lycopodan-5-one, 12-hydroxy-15-methyl-, (12.alpha.,15R)- | [C_16_H_25_NO_2_](https://pubchem.ncbi.nlm.nih.gov/#query=C16H25NO2) |  | 0 | 7.288 |
| 15 | 13.5858 | 1H-Indole-2-carboxylic acid, 6-(4-ethoxyphenyl)-3-methyl-4-oxo-4,5,6,7-tetrahydro-, isobutyl ester | C_21_H_25_ | 355.427 | 0.3288 | 0 |
| 16 | 13.849 | Octadecane | C_18_H_38_ | 254.4943 | 1.0234 | 0 |
| 17 | 14.1007 | Octacosane | C_28_H_58_ | 394.7601 | 1.4274 | 0 |
| 18 | 14.2838 | 1-Hexanol, 2-ethyl-2-propyl- | C_11_H_24_O | 172.313 | 0.4338 | 0 |
| 19 | 14.9876 | Heptasiloxane, hexadecamethyl- | C_16_H_48_O_6_Si_7_ | 533.1472 | 0.4445 | 1.289 |
| 20 | 15.8288 | Cyclodecasiloxane, eicosamethyl- | C_20_H_60_O_10_Si_10_ | 741.54 | 1.3971 | 0 |
| 21 | 16.4468 | 3,6-Dioxa-2,4,5,7-tetrasilaoctane, 2,2,4,4,5,5,7,7-octamethyl- | C_10_H_30_O_2_Si_4_ | 294.686 | 1.0423 | 0 |
| 22 | 17.1961 | 1,1,1,5,7,7,7-Heptamethyl-3,3-bis(trimethylsiloxy)tetrasiloxane | C_16_H_48_O_6_Si_7_ | 533.147 | 0 | 0.8063 |
| 23 | 17.8027 | Piperidine, 1-(5-trifluoromethyl-2-pyridyl)-4-(1H-pyrrol-1-yl)- | C_12_H_13_F_3_N_2_O_2_ | 274.24 | 0 | 6.1505 |
| 24 | 17.9859 | Heptadecane, 9-octyl- | C_18_H_38_ | 254.4943 | 0.7997 | 0 |
| 25 | 19.0388 | Hexasiloxane, tetradecamethyl- | C_14_H_42_O_5_Si_6_ | 458.993 | 10.9144 | 11.419 |
| 26 | 19.7024 | Pentacosane | C_25_H_52_ | 352.68 | 0 | 5.181 |
| 27 | 19.714 | Hexadecane, 2-methyl- | C_17_H_36_ | 240.467 | 1.8055 | 0 |
| 28 | 20.1258 | 1-Monopalmitin, 2TMS derivative | C_25_H_54_O_4_Si_2_ | 474.86 | 0 | 3.7148 |
| 29 | 20.6408 | 2,5-Dihydroxybenzoic acid, 3TMS derivative | C_16_H_30_O_4_Si_3_ | 370.66 | 0 | 0.5604 |
| 30 | 20.99 | Eicosane | C_20_H_42_ | 282.54 | 1.6745 | 4.2381 |
| 31 | 21.2759 | 2-Monostearin, 2TMS derivative | C_27_H_58_O_4_Si_2_ | 502.91 | 0 | 2.761 |
| 32 | 21.5162 | 6H-Benzo[g]-1,3-benzodioxolo[5,6-a]quinolizine-12-methanol, 5,8,13,13a-tetrahydro-10,11,14-trimethoxy-, (S)- | C_21_H_23_NO_5_ | 369.4 | 0 | 6.9054 |
| 33 | 22.1058 | 4-Hydroxybenzyl alcohol, 2TBDMS derivative | C_13_H_24_O_2_Si_2_ | 268.49 | 2.2367 | 0 |
| 34 | 22.2488 | Docosane | C_22_H_46_ | 310.6 | 8.3468 | 0 |
| 35 | 22.3345 | Hentriacontane | C_31_H_64_ | 436.84 | 0 | 1.2026 |
| 36 | 22.6721 | Hexacosane, 1-iodo- | C_26_H_53_I | 492.6035 | 0 | 0.9615 |
| 37 | 22.6837 | Tetracosane, 1-iodo- | C_24_H_49_I | 464.55 | 2.0782 | 0 |
| 38 | 23.542 | Nonacosane | C_29_H_60_ | 408.786 | 12.1253 | 0 |
| 39 | 23.7878 | 2-Quinolinecarboxylic acid, 6-bromo-1,4-dihydro-4-oxo-, ethyl ester | [C_11_H_8_BrNO_3_](https://pubchem.ncbi.nlm.nih.gov/#query=C11H8BrNO3) | 296.12 | 0.4549 | 2.1063 |
| 40 | 23.7995 | Heneicosane, 11-decyl- | C_31_H_64_ | 436.839 | 2.1744 | 0 |
| 41 | 23.9996 | Heptadecane, 3-methyl- | C_18_H_38_ | 254.4943 | 0 | 3.5848 |
| 42 | 24.0913 | Cyclononasiloxane, octadecamethyl- | C_18_H_54_O_9_Si_9_ | 667.3855 | 17.3937 | 0 |
| 43 | 25.0869 | Octadecane, 3-methyl- | C_19_H_40_ | 268.5209 | 3.4777 | 1.2225 |
| 44 | 25.4932 | Heneicosane | C_21_H_44_ | 296.5741 | 11.503 | 2.984 |
| 45 | 26.5744 | Hexadecane, 7,9-dimethyl- | C_18_H_38_ | 254.494 | 0 | 0.7776 |

**Supplementary Table S4** Phytoconstituent identified by GC-MS analysis of *H. salicornicum* chloroform extract. R.T.=Retention Time; MF=Molecular formula; M. wt. = Molecular weight.

| PK | RT | Library/ID | MF | M.wt | Haloxylon 1 | Haloxylon 2 | |
| --- | --- | --- | --- | --- | --- | --- | --- |
| 1 | 8.6591 | Cyclopentasiloxane, decamethyl- | C_10_H_30_O_5_Si_5_ | 370.7697 | 0 | 0.6324 |  |
| 2 | 8.9223 | Trisiloxane, 1,1,1,5,5,5-hexamethyl-3,3-bis[(trimethylsilyl)oxy]- | C_12_H_36_O_4_Si_5_ | 384.839 | 0 | 0.2825 |  |
| 3 | 8.9336 | Pentasiloxane, dodecamethyl- | C_12_H_36_O_4_Si_5_ | 384.8393 | 1.3176 | 1.1404 |  |
| 4 | 9.7805 | Glycerol, 3TMS derivative | C_12_H_32_O_3_Si_3_ | 308.6372 | 1.8333 | 0.4345 |  |
| 5 | 9.9521 | Silanol, trimethyl-, phosphate (3:1) | C_9_H_27_O_4_PSi_3_ | 314.5385 | 1.4675 | 0 |  |
| 6 | 10.1068 | 4'-Hydroxy-3'-methoxyacetophenone, trifluoroacetate | C_11_H_9_F_3_O_4_ | 262.18 | 0 | 0.3222 |  |
| 7 | 10.1124 | 2-Amino-4-(pyridin-3-yl)-4H-benzo[h]chromene-3-carbonitrile | [C_20_H_14_N_2_O](https://pubchem.ncbi.nlm.nih.gov/#query=C20H14N2O) | 298.3 | 1.0743 | 0 |  |
| 8 | 10.2956 | 1H-Indene, 2-phenyl- | C_15_H_12_ | 192.2558 | 0 | 1.2052 |  |
| 9 | 10.3699 | 5,6-Dimethoxy-1-indanone | C_11_H_12_O_3_ | 192.21 | 3.5908 | 0 |  |
| 10 | 10.7761 | Benzo[b]thiophene-4-acetic acid | [C_10_H_8_O_2_S](https://pubchem.ncbi.nlm.nih.gov/#query=C10H8O2S) | 192.24 | 1.6776 | 0 |  |
| 11 | 10.7819 | 5-Ethynyl-7-methoxy-2H-1,3-benzodioxol-4-ol | C_16_H_12_O_5_ | 284.26 | 0 | 0.5448 |  |
| 12 | 11.2282 | 2-Bromo dodecane | C_12_H_25_Br | 249.231 | 0 | 0.7626 |  |
| 13 | 11.2224 | Eicosane, 1-iodo- | C_20_H_41_I | 408.444 | 2.0784 | 0.3831 |  |
| 14 | 11.5829 | Benzoic acid, 2-[[(4,5-dimethyl-4H-1,2,4-triazol-3-yl)thio]methyl]- | C_11_H_11_N_3_O_2_ | 217.23 | 3.3108 | 0 |  |
| 15 | 11.5773 | 2,6-Bis(tert-butyl)phenol, TMS derivative | C_14_H_22_O | 206.32 | 0 | 2.304 |  |
| 16 | 11.9549 | 1-Phenyl-1-(trimethylsilyloxy)ethylene | C_11_H_16_OSi | 192.32 | 0 | 0.1998 |  |
| 17 | 12.0464 | Disulfide, di-tert-dodecyl | C_24_H_50_S_2_ | 402.8 | 0.6837 | 0 |  |
| 18 | 12.4985 | 2-Amino-2-oxo-acetic acid, N-[3,4-dimethylphenyl]-, ethyl ester | C_12_H_15_NO_3_ | 221.252 | 0 | 1.3469 |  |
| 19 | 12.7789 | Octadecane | C_18_H_38_ | 254.4943 | 0 | 0.5435 |  |
| 20 | 12.9619 | D-(-)-Tagatofuranose, pentakis(trimethylsilyl) ether (isomer 2) | C_21_H_52_O_6_Si_5_ | 541.06 | 0.3327 | 0 |  |
| 21 | 13.2709 | D-(-)-Fructofuranose, pentakis(trimethylsilyl) ether (isomer 2) | C_21_H_52_O_6_Si_5_ | 541.0615 | 0.8538 | 0 |  |
| 22 | 13.1222 | Cyclononasiloxane, octadecamethyl- | C_18_H_54_O_9_Si_9_ | 667.3855 | 0 | 0.0795 |  |
| 23 | 13.5685 | Piperidine, 1-(5-trifluoromethyl-2-pyridyl)-4-(1H-pyrrol-1-yl)- | C_15_H_16_F_3_N_3_ | 295.30 | 0 | 1.6501 |  |
| 24 | 13.7974 | Heptadecane, 3-methyl- | 254.4943 | 0.3436 | 0 | 0.1988 |  |
| 25 | 13.9575 | .beta.-D-(+)-Talopyranose, 5TMS derivative | C_21_H_52_O_6_Si_5_ | 541.06 | 0.4865 | 0 |  |
| 26 | 14.0663 | Heptadecane | C_17_H_36_ | 240.4677 | 1.0464 | 0 |  |
| 27 | 14.4612 | 3-Indolylacetamide, TMS derivative | C_16_H_26_N_2_OSi_2_ | 318.5614 | 0 | 32.3949 |  |
| 28 | 14.4725 | Hexadecane, 1-chloro- | C_16_H_33_Cl | 260.88 | 2.3516 | 0 |  |
| 29 | 14.8158 | .alpha.-D-Mannopyranose, 5TMS derivative | C_21_H_52_O_6_Si_5_ | 541.06 | 0.6246 | 0 |  |
| 30 | 15.3079 | Palmitic Acid, TMS derivative | C_19_H_40_O_2_Si | 328.6052 | 4.2243 | 5.4822 |  |
| 31 | 16.458 | Heneicosane | C_27_H_56_ | 380.7335 | 0.6764 | 2.9079 |  |
| 32 | 16.4467 | 3,6-Dioxa-2,4,5,7-tetrasilaoctane, 2,2,4,4,5,5,7,7-octamethyl- | C_10_H_30_O_2_Si_4_ | 294.686 | 0 | 1.9768 |  |
| 33 | 16.9959 | 13-Octadecenoic acid, (E)-, TMS derivative | C_21_H_42_O_2_Si | 354.64 | 4.788 | 0 |  |
| 34 | 17.0189 | 9,12-Octadecadienoic acid (Z,Z)-, TMS derivative | C_21_H_40_O_2_Si | 352.62 | 2.7875 | 3.4405 |  |
| 35 | 17.1963 | Stearic acid, TMS derivative | C_21_H_44_O_2_Si | 356.6584 | 0 | 2.2338 |  |
| 37 | 17.6825 | benzaldehyde, 4-(5-nitro-2-benzoxazolyl)- | C_12_H_14_N_2_O_3_ | 234.25 | 2.9688 | 0 |  |
| 38 | 17.6769 | Hexadecane, 2,6,10,14-tetramethyl- | C_20_H_42_ | 282.5475 | 0 | 0.3486 |  |
| 39 | 17.9802 | Docosane, 1-iodo- | C_22_H_45_I | 436.4972 | 0 | 0.7054 |  |
| 40 | 18.4494 | 2,5-Dihydroxybenzoic acid, 3TMS derivative | C_16_H_30_O_4_Si_3_ | 370.66 | 0 | 0.2002 |  |
| 41 | 19.0387 | Heptasiloxane, hexadecamethyl- | C_16_H_48_O_6_Si_7_ | 533.1472 | 0 | 0.5441 |  |
| 42 | 19.0272 | 2-Propenoic acid, 3-(4-methoxyphenyl)-, 2-ethylhexyl ester | C_18_H_26_O_3_ | 290.3972 | 0.524 | 0 |  |
| 43 | 19.1932 | pyridine, 2,6-diphenyl-4-(1-piperidinyl)- | [C_17_H_17_NO](https://pubchem.ncbi.nlm.nih.gov/#query=C17H17NO) | 251.32 | 0 | 0.3382 |  |
| 44 | 19.3305 | Norgestrel | C_21_H_28_O_2_ | 312.453 | 4.9748 | 0 |  |
| 45 | 19.4278 | Hexadecane, 2,6,10,14-tetramethyl- | C_20_H_42_ | 282.5475 | 0 | 0.4316 |  |
| 46 | 19.7082 | Tetracosane | C_24_H_50_ | 338.65 | 0 | 1.2438 |  |
| 47 | 19.7367 | Hexacosane | C_26_H_54_ | 366.707 | 1.5567 | 0 |  |
| 48 | 20.1144 | 1-Monopalmitin, 2TMS derivative | C_25_H_54_O_4_Si_2_ | 474.8649 | 4.564 | 0 |  |
| 49 | 20.2919 | Docosane | C_22_H_46_ | 310.6 | 0 | 0.1907 |  |
| 50 | 20.6523 | 6,6-Diethylhoctadecane | C_20_H_42_ | 282.5475 | 0 | 0.4312 |  |
| 51 | 20.7095 | Sucrose, 8TMS derivative | C_36_H_86_O_11_Si_8_ |  | 0.455 | 0 |  |
| 52 | 20.8353 | Tricosane | 919.7454 |  | 1.2602 | 0 |  |
| 53 | 21.522 | Glycerol monostearate, 2TMS derivative | C_27_H_58_O_4_Si_2_ | 502.9180 | 2.5273 | 0 |  |
| 54 | 21.5278 | 6H-Benzo[g]-1,3-benzodioxolo[5,6-a]quinolizine-12-methanol, 5,8,13,13a-tetrahydro- | C_21_H_23_NO_5_ | 369.4 | 0 | 2.7315 |  |
| 55 | 21.894 | (4-Benzooxazol-2-ylpiperazin-1-yl)(tetrahydrofuran-2-yl)methanone | C_16_H_19_N_3_O_3_ | 301.142 | 0 | 1.4501 |  |
| 56 | 21.8481 | Squalene | C_30_H_50_ | 410.7 | 1.5633 | 0 |  |
| 57 | 22.3174 | Methoxyacetic acid, 2-tridecyl ester | C_16_H_32_O_3_ | 272.42 | 0 | 2.2313 |  |
| 58 | 22.5692 | Hexacosane, 1-iodo- | C_24_H_49_I | 464.55 | 0 | 2.3994 |  |
| 59 | 22.5348 | Cyclohexane, 1,2,4,5-tetraethyl- | C_10_H_20_ | 140.265 | 1.8563 | 0 |  |
| 60 | 22.735 | Octadecane, 3-methyl- | C_19_H_40_ | 268.5209 | 2.0922 | 2.9599 |  |
| 61 | 23.1185 | Thiazole, 2,4-dihydroxy- | C_3_H_3_NO_2_S | 117.13 | 0 | 3.5436 |  |
| 62 | 23.1756 | Silane, trichlorooctadecyl- | C_18_H_37_Cl_3_Si | 387.93 | 0.6535 | 0 |  |
| 63 | 23.5132 | Nonacosane | C_29_H_60_ | 408.786 | 3.1469 | 0 |  |
| 64 | 23.7937 | Hexasiloxane, tetradecamethyl- | C_14_H_42_O_5_Si_6_ | 458.993 | 0 | 6.649 |  |
| 65 | 23.7993 | Eicosane | C_20_H_42_ | 282.54 | 7.3554 | 1.0483 |  |
| 66 | 23.9882 | Methoxyacetic acid, 3-pentadecyl ester | C_19_H_36_O_4_ | 328.48 | 2.2205 | 0 |  |
| 67 | 23.9997 | Octacosane | C_28_H_58_ | 394.7601 | 0 | 3.1962 |  |
| 68 | 24.1083 | Cholest-5-en-3-ol, (3.alpha.)-, TMS derivative | C_30_H_54_OSi | 458.83 | 2.2942 | 0 |  |
| 69 | 24.4059 | 3-Cyclohexene-1-ethanol, .alpha.-ethenyl-.alpha.,3-dimethyl-6-(1-methylethylidene)- | C_15_H_24_O | 220.35 | 6.5179 | 0 |  |
| 70 | 24.4346 | Pentacosane | C_25_H_52_ | 352.6804 | 0 | 2.9931 |  |
| 71 | 24.5948 | Hexadecane, 6,11-dipentyl- | C_26_H_54_ | 366.70 | 0 | 0.8795 |  |
| 72 | 25.0124 | Stigmasterol, TMS derivative | C_32_H_56_OSi | 484.8719 | 3.539 | 0 |  |
| 73 | 25.4358 | Stigmast-5-ene, 3.beta.-(trimethylsiloxy)-, (24S)- | C_32_H_58_OSi | 486.8878 | 6.6387 | 0 |  |
| 74 | 25.9565 | 1,4-Methanoazulen-9-ol, decahydro-1,5,5,8a-tetramethyl-, [1R (1.alpha.,3a.beta.,4.alpha.,8a.beta.,9S*)]- | C_15_H_26_O | 222.36 | 0.8281 | 0 |  |
| 75 | 26.5746 | 2-Quinolinecarboxylic acid, 6-bromo-1,4-dihydro-4-oxo-, ethyl ester | C_12_H_10_BrNO_3_ | 296.12 | 0 | 0.5198 |  |
| 76 | 27.696 | 2-Trifluoroacetoxytridecane | [C_14_H_25_F_3_O_2_](https://pubchem.ncbi.nlm.nih.gov/#query=C14H25F3O2) | 282.34 | 0.3931 | 0 |  |

**Supplementary Table S5** Plus/Minus Data For SDS-PAGE Gel Image. The fractionation of TCPs extracted from the eight studied plant taxa were analyzed and showed Total protein bands, Polymorphic bands (including unique bands), Polymorphic bands (without including unique bands), Unique protein bands as revealed by the analysis of protein profiles using SDS-PAGE technique. RF denotes for rate of flow. MW refers to detected molecular weight.

| **RF** | **MW** | ***T. aphylla*** | | ***Z. coccineum*** | | ***E. glaucophyllum*** | | ***H. salicornicum*** | | **Frequency** | **Polymorphism** |
| --- | --- | --- | --- | --- | --- | --- | --- | --- | --- | --- | --- |
|  |  | **L1** | **L2** | **L1** | **L2** | **L1** | **L2** | **L1** | **L2** |  |  |
| 0.093 | 140.300 | - | + | - | - | - | - | - | - | 0.125 | Unique |
| 0.113 | 134.166 | - | - | - | - | + | + | + | + | 0.500 | Polymorphic |
| 0.119 | 132.378 | - | - | + | - | - | - | - | - | 0.125 | Unique |
| 0.152 | 122.964 | + | + | + | + | + | + | + | + | 1.000 | Monomorphic |
| 0.164 | 119.709 | - | + | - | - | - | - | - | - | 0.125 | Unique |
| 0.185 | 114.220 | - | + | + | - | - | - | - | - | 0.250 | Polymorphic |
| 0.211 | 107.770 | + | + | + | + | + | + | + | + | 1.000 | Monomorphic |
| 0.238 | 101.458 | - | - | - | + | - | - | - | - | 0.125 | Unique |
| 0.241 | 100.780 | - | - | - | - | + | + | + | + | 0.500 | Polymorphic |
| 0.245 | 99.883 | + | + | - | - | - | - | - | - | 0.250 | Polymorphic |
| 0.254 | 97.893 | - | - | + | - | - | - | - | - | 0.125 | Unique |
| 0.260 | 96.589 | - | - | - | - | + | + | - | - | 0.250 | Polymorphic |
| 0.278 | 92.780 | - | - | + | - | - | - | - | - | 0.125 | Unique |
| 0.284 | 91.544 | - | - | - | - | + | + | - | - | 0.250 | Polymorphic |
| 0.321 | 84.277 | + | + | + | + | + | + | + | + | 1.000 | Monomorphic |
| 0.359 | 77.413 | + | + | - | - | - | - | - | - | 0.250 | Polymorphic |
| 0.373 | 75.028 | - | - | - | + | + | + | + | + | 0.625 | Polymorphic |
| 0.411 | 68.918 | + | + | + | + | + | + | + | + | 1.000 | Monomorphic |
| 0.454 | 62.602 | - | - | - | - | - | + | + | + | 0.375 | Polymorphic |
| 0.456 | 62.322 | - | - | - | + | - | - | - | - | 0.125 | Unique |
| 0.489 | 57.890 | + | + | + | - | - | - | - | - | 0.375 | Polymorphic |
| 0.492 | 57.503 | - | - | - | - | - | + | - | - | 0.125 | Unique |
| 0.512 | 54.989 | - | - | - | - | - | - | + | + | 0.250 | Polymorphic |
| 0.542 | 51.422 | - | - | - | - | - | + | - | - | 0.125 | Unique |
| 0.546 | 50.965 | + | + | + | - | - | - | - | - | 0.375 | Polymorphic |
| 0.575 | 47.765 | - | - | + | + | - | - | - | - | 0.250 | Polymorphic |
| 0.582 | 47.024 | - | - | - | - | - | + | + | + | 0.375 | Polymorphic |
| 0.608 | 44.369 | - | - | - | + | - | - | - | - | 0.125 | Unique |
| 0.684 | 37.436 | + | + | + | + | + | + | + | + | 1.000 | Monomorphic |
| 0.729 | 33.854 | + | + | + | + | + | + | + | + | 1.000 | Monomorphic |
| 0.765 | 31.236 | - | - | + | + | - | - | - | - | 0.250 | Polymorphic |
| 0.770 | 30.889 | - | - | - | - | - | + | + | + | 0.375 | Polymorphic |
| 0.812 | 28.121 | - | + | + | + | + | + | + | + | 0.875 | Polymorphic |
| 0.847 | 26.004 | - | - | + | + | - | - | - | - | 0.250 | Polymorphic |
| 0.888 | 23.727 | + | + | + | + | + | + | + | + | 1.000 | Monomorphic |
| 0.926 | 21.795 | + | + | + | + | + | + | + | + | 1.000 | Monomorphic |
| 0.969 | 19.797 | + | + | + | + | + | + | + | + | 1.000 | Monomorphic |
| 0.986 | 19.059 | + | + | + | + | + | + | + | + | 1.000 | Monomorphic |


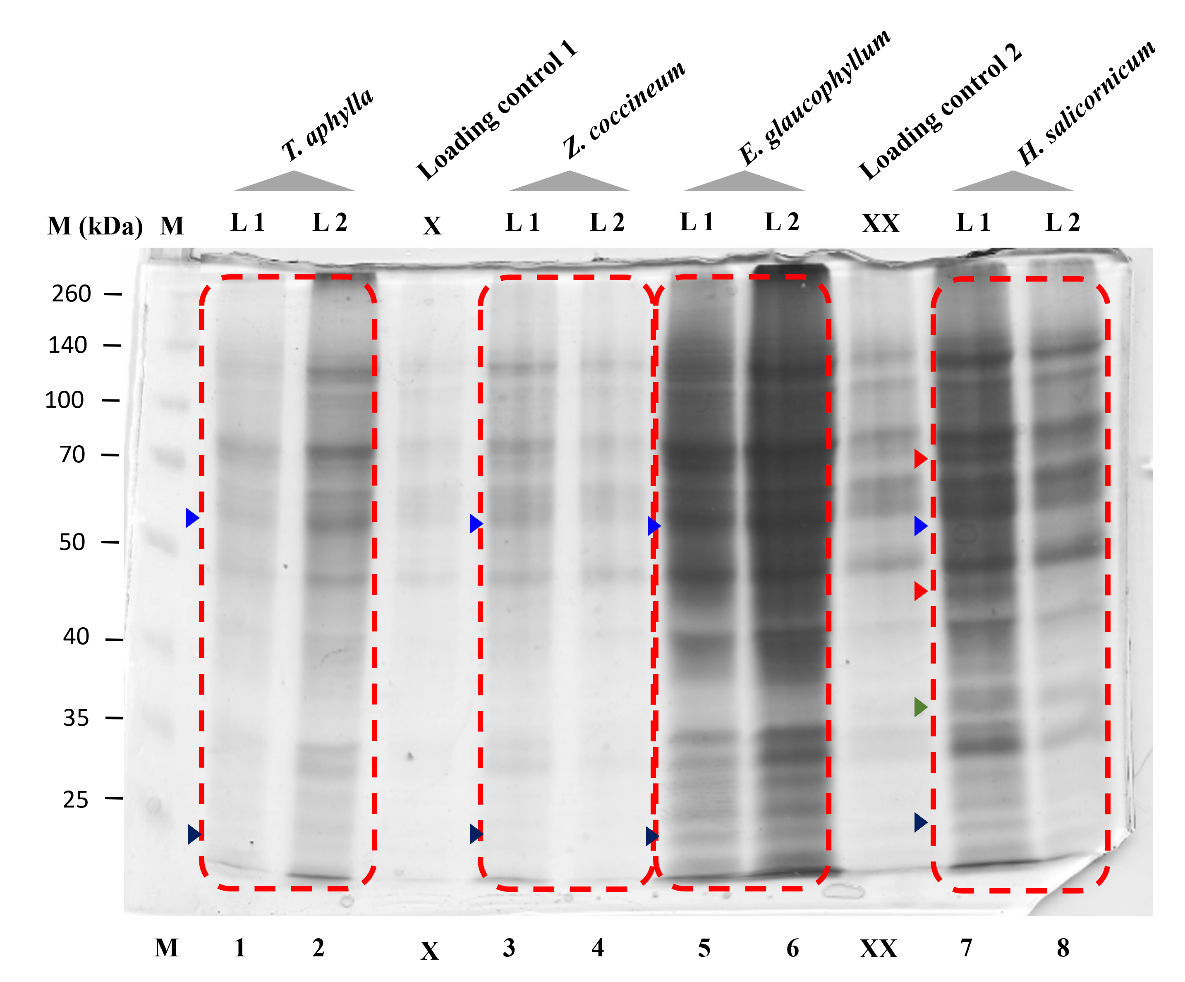


**Supplementary Fig. S1** Original full length, Uncropped, and unprocessed electrophoretic banding patterns of extracted total cellular proteins (TCPs) of the four studied plant species from the two different locations (Al-Qalyubia (L1) and Al-Suez (L2) Governorates) using SDS-PAGE technique. Lane M: protein marker (Spectra™ Multicolor Broad Range Protein Ladder, Thermo Scientific™, Cat. number 26634), Lane 1: *T. aphylla* L1, Lane 2: *T. aphylla* L2, Lane 3: *Z. coccineum* L1, Lane 4: *Z. coccineum* L2, Lane 5: *E. glaucophyllum* L1, Lane 6: *E. glaucophyllum* L2, Lane 7: *H. salicornicum* L1, Lane 8: *H. salicornicum* L2. Lane X, XX: refer to loading control 1 and 2 (20 µg and 40 µg total protein content, respectively of *T. aphylla* for example) to manifest the homogeneity of extracted TCPS. The protein gel was stained with CoBB stain. The numbers shown on the left-handed side of the figure refer to molecular weight standards in kDa (Spectra™ Multicolor Broad Range Protein Ladder). Red arrowheads refer to pronounced polypeptides detected in *H. salicornicum* L1, but barely or not detected in L2 sample. Light and dark blue arrowheads refer to approximate molecular weights of RuBisCO large (RbcLS) and small (RbcSS) subunits, respectively. Green arrowhead refers to a unique protein band running approximately at 30 kDa was detected in *H. salicornicum* (L1), but not detected in the rest of samples. Full-length uncropped and unprocessed gel accompanied this manuscript as Supplementary Information File and was presented in Supplementary Figure (Fig. S1). Red dotted rectangles refer to the positions where the cropped was executed.


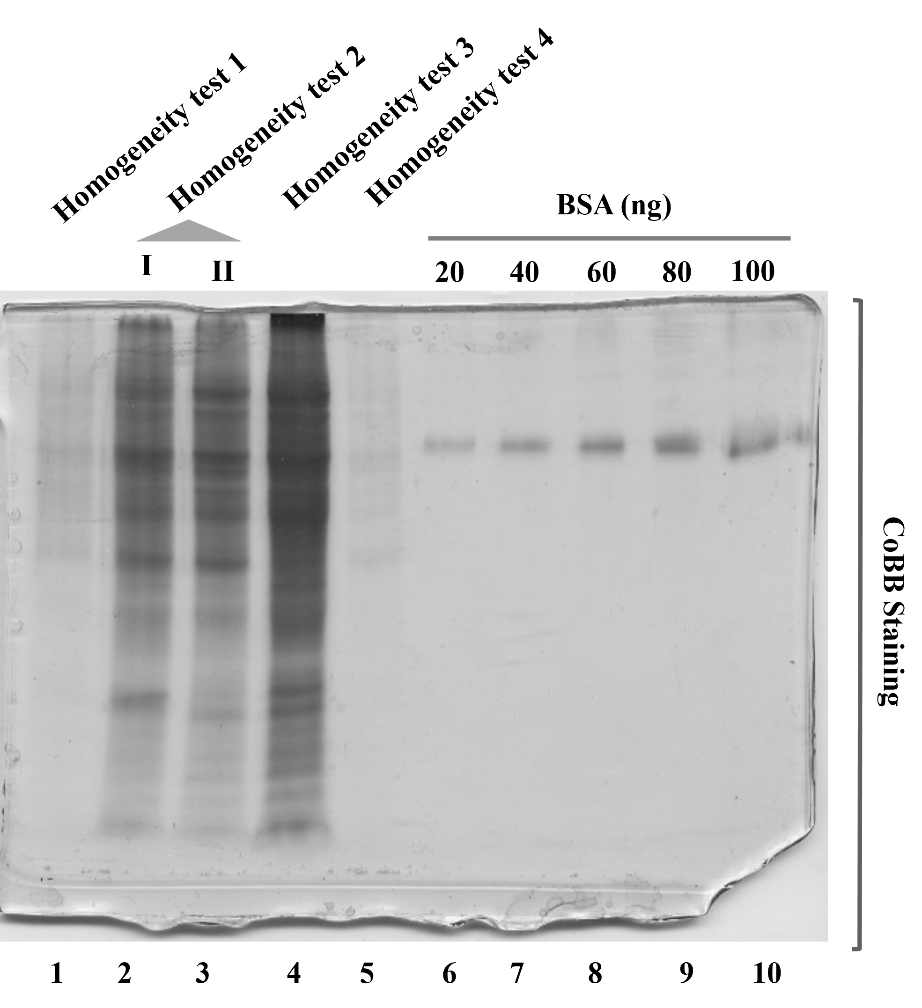


**Supplementary Fig. S2** Original full length, Uncropped, and unprocessed analytical SDS-PAGE manifested the homogeneity of extracted total cellular proteins (TCPs) of the four studied plant species from the two different locations (Al-Qalyubia (L1) and Al-Suez (L2) Governorates). Electrophoretic banding patterns of differently loaded concentration of TCPs of *E. glaucophyllum* (another example to manifest integrity and extraction homogeneity) of Lane 1: 20 µg, Lane 2-3: duplicate loading (I and II) of 60 µg, Lane 4: 80 µg, Lane 5: 10 µg. Lanes 6-10: Electrophoretic running of ascending concentration series from 20 to 100 ng respectively of BSA (Bovine serum albumin as protein size standard) to quantify RuBisCO_LS_ concentration in (ng). The protein gel was stained with CoBB stain. Full-length uncropped and unprocessed gel accompanied this manuscript as Supplementary Information File and was presented in Supplementary Figure (Fig. S2).


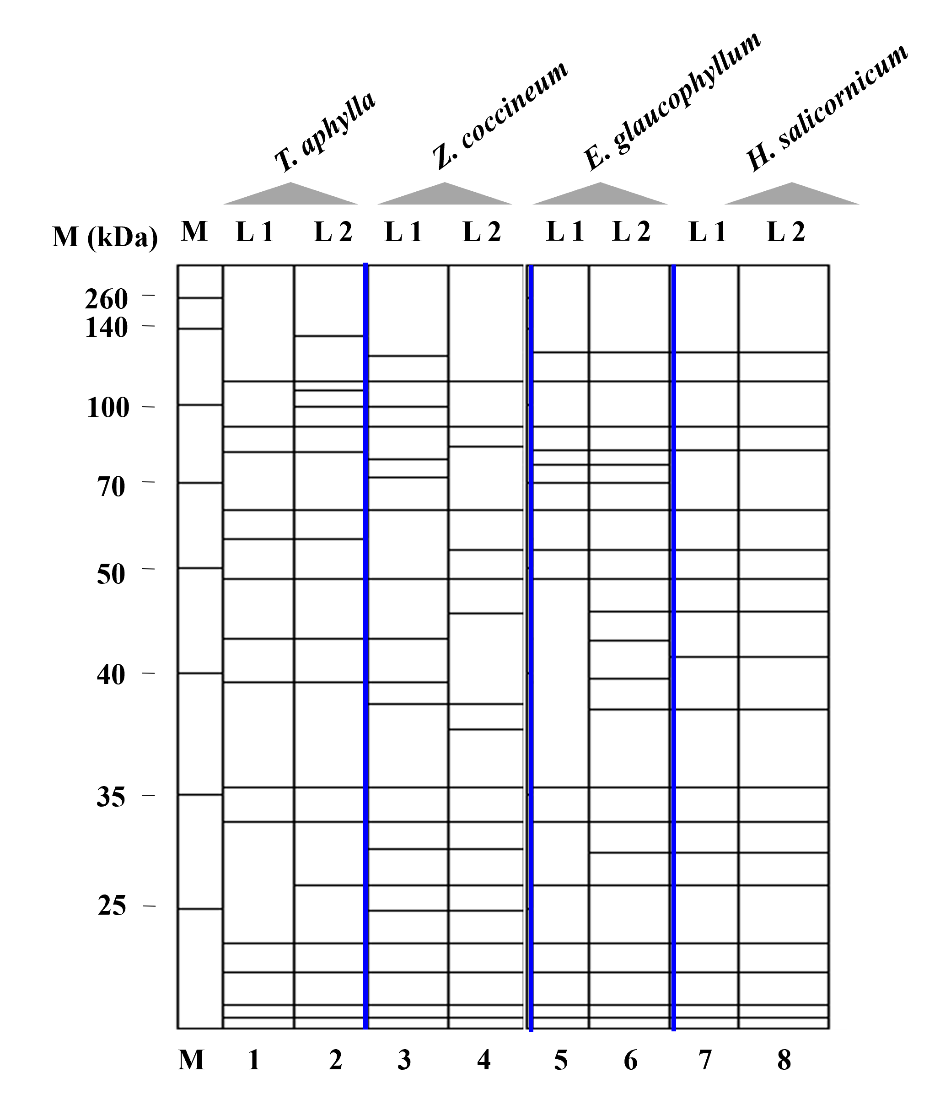


**Supplementary Fig. S3** Linear schematic representation of executed band scoring was shown. The SDS-PAGE protein profiles and band scoring revealed a total of 38 protein bands with polymorphism percentage (P % = 73.68 %) of 10 monomorphic and 28 polymorphic bands (Fig. 10a, Table S1). TCPs were extracted from the eight studied taxa belonging to four plant species (listed in Table 1) at the vegetative stage. The protein banding patterns of 100 µg TCPs (equivalent to total protein content) unless otherwise stated were separated using 12% SDS-PAGE technique (Fig. 9). The numbers shown on the left-handed side of the figure refer to molecular weight standards in kDa (Spectra™ Multicolor Broad Range Protein Ladder).
